# Dual-metal-organic framework and gallic acid incorporated 3D-printed scaffolds: Revolutionizing refractory bone defect repair through immune-angiogenic-neurogenic synergy

**DOI:** 10.1016/j.mtbio.2025.102323

**Published:** 2025-09-19

**Authors:** Yongbo Li, Yifei Guo, Yuanpei Cheng, Xiaodong Liu, Hengren Li, Chen Liu, Xipeng Chen, Heng Yang, Xingzhi Jing, Xiaoyang Liu, Han Wu, Min Guo, Peibiao Zhang, Xingang Cui

**Affiliations:** a Department of Spine Surgery, Shandong Provincial Hospital Affiliated to Shandong First Medical University, Jinan, 250000, Shandong, PR China; b State Key Laboratory of Polymer Science and Technology, Changchun Institute of Applied Chemistry, Chinese Academy of Sciences, Changchun, 130022, PR China; c Department of Orthopedics, China-Japan Union Hospital of Jilin University, Changchun, 130000, PR China

**Keywords:** Metal-organic frameworks, 3D printing scaffold, Gallic acid, Refractory bone defect, Neurogenesis, Multicellular modulation

## Abstract

Steroid-associated osteonecrosis (SAON)-related bone defects are refractory and present significant therapeutic challenges due to dysregulated multiple cellular functions and disrupted multidimensional microenvironments. Despite progress in regulating immune responses and promoting vascularization for SAON-related bone defects, effective neural innervation strategies remain limited. Notably, immune response, angiogenesis, and neural innervation are interdependent processes that collectively regulate bone regeneration. Herein, we engineered a novel 3D-printed composite scaffold with highly interconnected porosity and multiple bioactivities by integrating magnesium-copper dual-metal-organic framework (MgCu-MOF74), gallic acid (GA) and polylactic acid (PLA). MgCu-MOF74 exhibits antioxidant capacity, controllable release of metal ions, and osteo-angiogenic properties. The composite scaffold demonstrated excellent mechanical properties and degradation characteristics well suited for bone regeneration. More importantly, the incorporation of GA and dual-ion synergy enabled the scaffold to achieve pronounced multicellular modulation by promoting macrophage polarization, inducing endothelial cell-mediated angiogenesis, stimulating Schwann cell morphological maturation, and enhancing the osteogenic differentiation of bone marrow-derived mesenchymal stem cells (BMSCs), while markedly increasing intercellular crosstalk to optimize the local multidimensional microenvironment. In vivo studies further confirmed that the scaffold effectively facilitates the repair of SAON-related bone defects by harnessing the synergistic interactions among the immune, angiogenic, and neurogenic microenvironments. This work provides an innovative strategy for treating refractory SAON - related bone defects, highlighting the potential of the developed scaffold in modulating diverse cell types and remodeling complex microenvironments.

## Highlights

• This study first shows GA regulates neuronal activity/axonal growth via MAPK/ERK pathway.
• MgCu-MOF74 has antioxidant capacity, controllable metal ion release, and osteo-angiogenic properties.
• Scaffold restores mitochondrial function (oxidative stress) and directs macrophage anti-inflammatory polarization.
• Gallic acid and dual-ion synergy modulates cellular behaviors, enhances crosstalk, optimizes microenvironment.
• Scaffold induces neovascularization and osteogenesis via PI3K/Akt pathway.

## Graphical abstract

**Figure:**
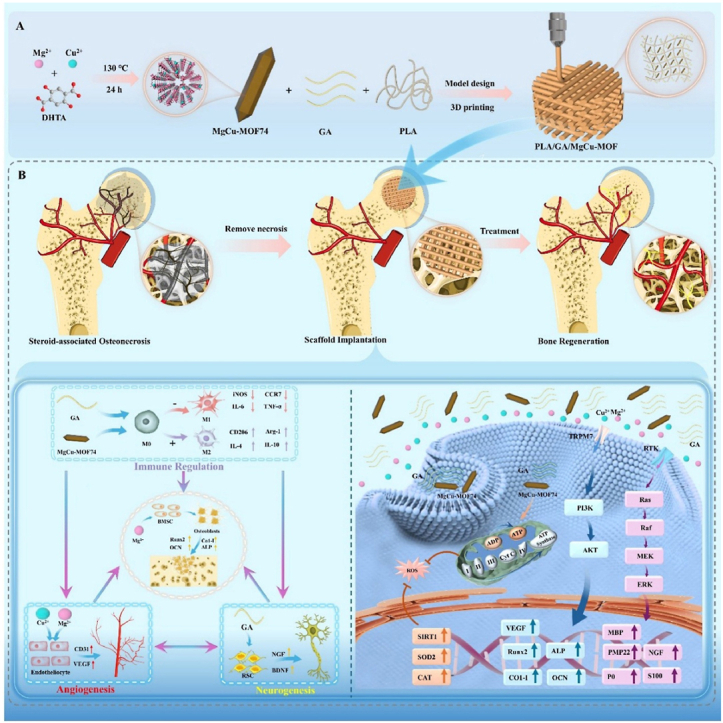

## 1 Introduction

Osteonecrosis due to long-term glucocorticoid use is a common debilitating condition in orthopedics [1]. As an early intervention strategy, core decompression (CD) removes necrotic bone tissue and alleviates intraosseous pressure to slow disease progression [2]. However, the resulting osseous defects necessitate filling materials to maintain local mechanical strength and prevent bone collapse [3]. Although autologous bone grafts are considered the gold standard following CD, their limited availability, high cost, and donor site morbidity restrict their broader application [4]. To overcome these limitations, personalized scaffolds fabricated via 3D printing technology have recently emerged owing to their high-precision customization and superior mechanical properties [5]. However, the complex osteonecrotic microenvironment significantly limits the effectiveness of biomaterial scaffolds, as such approaches cannot simultaneously address multiple cellular dysfunctions and multidimensional microenvironmental injuries.

Glucocorticoids can induce oxidative stress in cells within osteonecrotic regions, causing mitochondrial damage, extensive release of reactive oxygen species (ROS), and disruption of the endogenous antioxidant defense system [6,7]. This oxidative-antioxidative imbalance further contributes to osteoblast and endothelial cell dysfunction, alongside immune dysregulation. It not only exacerbates the inflammatory response but also reduces the expression of sensory neuropeptides (e.g., calcitonin gene-related peptide [CGRP], substance P [SP]) [8,9]. Consequently, these changes lead to vascular and neural injury, accelerating femoral head necrosis progression [10].

It is well known that macrophages are crucial for bone injury healing and biomaterial-induced immunomodulation [11,12]. Meanwhile, as a highly innervated tissue, bone exhibits an extensive distribution of nerve fibers throughout the periosteum, bone marrow, and mineralized regions, indicating a close interrelationship between bone growth and neural regulation [13,32]. Previous studies have demonstrated that bioactive molecules, including neurotransmitters, neuropeptides, axon guidance factors, and neurotrophic factors, can significantly promote the regeneration and repair of bone tissue [[14], [15], [16], [17], [18], [19]]. Moreover, the vascular system is vital for nutrient transport, metabolic waste removal, and the recruitment of osteoprogenitor cells to defect sites [20]. Notably, the vascularization process can enhance the early expression of neuropeptide receptors, facilitating neural regeneration and accelerating bone defect repair [21]. Although significant progress has been achieved in regulating immune responses and promoting vascularization for the treatment of steroid-associated osteonecrosis (SAON)-related bone defects [22,23], effective strategies for achieving neural innervation remain limited. It is important to recognize that immune response, angiogenesis, and neural innervation are interdependent processes that collectively regulate bone regeneration [24]. Previous bioactive materials have been limited in constructing an intrinsic bone regeneration microenvironment, primarily due to insufficient capability for coordinated regulation of multiple cell types. Thus, employing bioactive materials to modulate multiple cell types and achieve the synergistic effects of immune regulation, vascularization, and neural regeneration presents a promising strategy for treating SAON-related bone defects.

Metal-organic frameworks (MOFs), composed of metal ions and organic ligands, have attracted significant attention in biomedical research for their unique structures and multifunctionality [25,26]. Their porosity and biocompatibility make them promising for drug delivery [27,28], while released metal ions offer therapeutic benefits [29,30]. Among them, magnesium-based Mg-MOF-74 stands out for releasing Mg^2+^, a key ion in bone regeneration that promotes osteoblast function [15,31]. Mg^2+^ promotes osteoblast adhesion, proliferation, and differentiation, while also modulating inflammation and supporting angiogenesis [32]. However, the poor water stability of Mg-MOF-74 causes rapid ion release and toxicity, limiting clinical use. To solve this, doping with metal ions like Zn^2+^ and Cu^2+^ has been studied [19,33]. Cu^2+^, with high charge density, enhances MOF stability and, at low doses, boosts osteoblast activity and vascularization [34,35]. Notably, a previous study has identified mixed valences (Cu^+^/Cu^2+^) in Cu-doped bimetallic MOFs [33], yet their antioxidant properties remain unexplored. The rapid interconversion between Cu^2+^ and Cu^+^ is theoretically expected to scavenge excessive ROS to mitigate oxidative stress and exert antioxidant effects. Therefore, incorporating Cu^2+^ into Mg-MOF-74 can enhance the material's water stability and antioxidant capacity while maintaining its osteogenic and angiogenic capabilities. This dual-ion (Mg^2+^/Cu^2+^) synergy presents a promising strategy for bone tissue engineering.

Gallic acid (GA), a naturally occurring polyphenol present in fruits, nuts, tea, and wine, possesses robust antibacterial, anti-inflammatory, and antioxidant properties [36,37].Notably, GA exhibits superior free radical scavenging abilities compared to vitamin E and has shown protective effects against neurological, cardiovascular, and gastrointestinal disorders [[38], [39], [40]].While previous studies have reported the application of GA in neural repair and neurodegenerative diseases [41,42], its underlying mechanism and potential application in bone regeneration have not been fully elucidated. Based on the anti-inflammatory, antioxidant, and neuroprotective properties of GA, this study aims to integrate GA into scaffold materials to enhance their performance in promoting neural regeneration and osteogenesis.

**Scheme 1:**
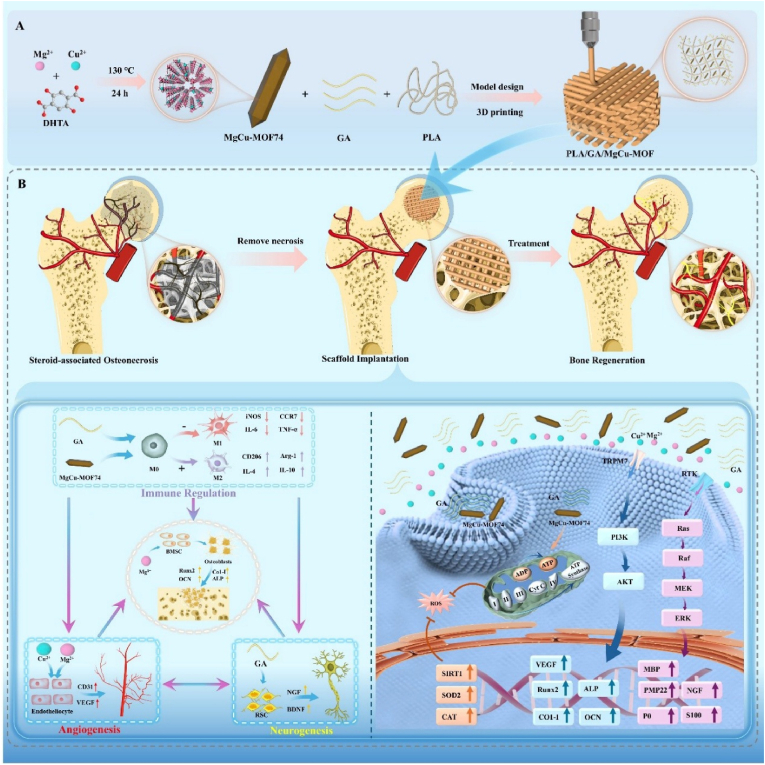
Schematic illustration of the dual-metal organic framework and gallic acid incorporated 3D-printed scaffold that effectively addresses refractory bone defect through the triple synergism of immune-angiogenic-neurogenic microenvironments.

In this study, the mechanisms underlying GA's effect on neurogenesis were explored. Subsequently, a water-stable, bimetallic metal–organic framework (MgCu-MOF74) was synthesized via a one-step hydrothermal method. The synthesized MgCu-MOF74 effectively scavenged ROS and allowed for a slow and sustained release of active ions, thereby avoiding the toxicity from ion burst release due to poor water stability. Following this, PLA/GA/MgCu-MOF scaffolds were fabricated by 3D printing using MgCu-MOF74 and GA as active components combined with poly (lactic acid) (PLA). The scaffolds exhibited excellent mechanical performance, degradability, and multifunctional bioactivity. Through the incorporation of GA and dual-ion synergy, the scaffolds modulated specific cellular behaviors and markedly enhanced intercellular crosstalk, thereby optimizing the local multidimensional microenvironment. In the early stage, the scaffolds regulate oxidative phosphorylation, upregulate antioxidant gene expression to restore mitochondrial function and enhance ATP generation under oxidative stress, and direct macrophage polarization toward an anti-inflammatory phenotype. In the mid-to-late phase, the scaffolds activate the MAPK/ERK and PI3K-Akt signaling pathways, stimulate Schwann cell maturation and axonal extension, induce angiogenesis, and promote osteoblastic differentiation (Scheme 1). This study presents a comprehensive repair strategy for SAON-related bone defects through synergistic immunomodulation, angiogenesis, and neurogenesis, offering new insights into the treatment of refractory bone defects.

## 2 Results and discussion

### 2.1 Effect of GA on neurogenesis

**Fig. 1:**
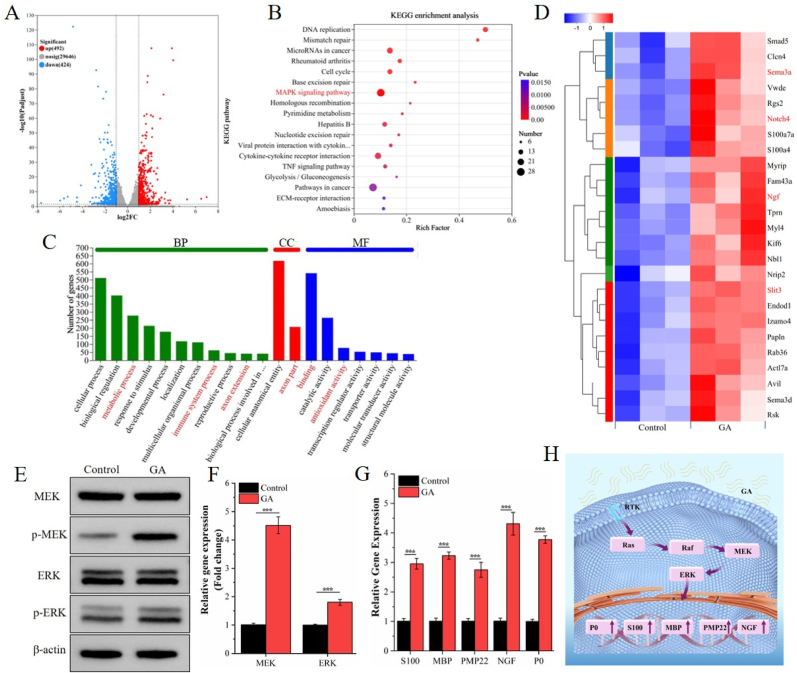
Effect of GA on neurogenesis. (A) Volcano plot of the differentially expressed genes (DEGs) between GA and Control groups. (B) Kyoto encyclopedia of genes and genomes (KEGG) enrichment analysis, (C) Gene Ontology (GO) analysis, and (D) A heatmap of DEGs. (E) Western blot analysis of MEK, p-MEK, ERK, and p-ERK. (F) Quantification of the p-MEK/MEK and p-ERK/ERK ratios. (G) RT‒qPCR analysis of S100, MBP, PMP22, NGF and P0 gene expression. (H) Schematic diagram showing the mechanism by which GA promotes neurogenesis. (n = 3; ∗*P* < 0.05, ∗∗*P* < 0.01, ∗∗∗*P* < 0.001).

To elucidate the mechanisms underlying GA's effect on neurogenesis, RNA sequencing was performed on RSC-96 cells treated with GA. The volcano plot showed 529 upregulated and 680 downregulated differentially expressed genes (DEGs) (Fig. 1A). Subsequent Kyoto Encyclopedia of Genes and Genomes (KEGG) enrichment analysis highlighted the mitogen-activated protein kinase (MAPK) signaling pathway (marked in red) as notably enriched in GA-treated cells (Fig. 1B). Additionally, Gene Ontology (GO) analysis identified enriched biological processes (metabolic processes, immune system processes, and axon extension), cellular components (axon part), and molecular functions (binding and antioxidant activity) (Fig. 1C, all marked in red**)**. These alterations are intimately associated with neurogenesis, providing critical insights into GA's modulation of neural development. Compared to the control group, GA-treated cells exhibited upregulation of key genes associated with neurogenesis, including Sema3a, Notch4, NGF, and Slit3 (Fig. 1D, marked in red). As axon guidance factors, Sema3a, secreted by specific neuronal subpopulations, and Slit3, predominantly expressed in sensory neurons, have been linked to angiogenesis and bone regeneration [13,43]. In addition, nerve growth factor (NGF) levels are significantly elevated in bone defect areas, accompanied by the development of bone-associated nerve fibers [24]. Recent research has also demonstrated NGF's essential role in vascular reconstruction and osteogenic differentiation during early bone repair [44,45]. Furthermore, the involvement of the Notch signaling pathway in neurogenesis regulation has been well-established in multiple studies [46,47]. Collectively, these results suggest that GA may enhance neurogenesis-related gene expression by activating MAPK-associated signaling pathways.

To further validate the biological mechanisms through which GA promotes neurogenesis, we conducted Western blot analysis to assess the expression of key proteins in the MAPK signaling pathway. Compared to the control group, GA-treated cells showed significantly higher phosphorylation levels of MEK and ERK (Fig. 1E and F). We then performed qRT-PCR on RSC-96 cells cultured for 5 days to evaluate gene expression and examine GA's impact on intracellular gene regulation. In comparison to the control group, GA therapy markedly increased a number of neurogenesis-related genes, such as S100, MBP (myelin basic protein), PMP22 (peripheral myelin protein), NGF, and P0 (myelin protein zero) (Fig. 1G).Collectively, these findings suggest that GA may regulate neuronal activity and axonal growth by activating the MAPK/ERK signaling pathway and upregulating neurogenesis-related gene expression (Fig. 1H), providing new insights into its role in modulating neurogenesis.

### 2.2 Synthesis and characterization of MgCu-MOF74

**Fig. 2:**
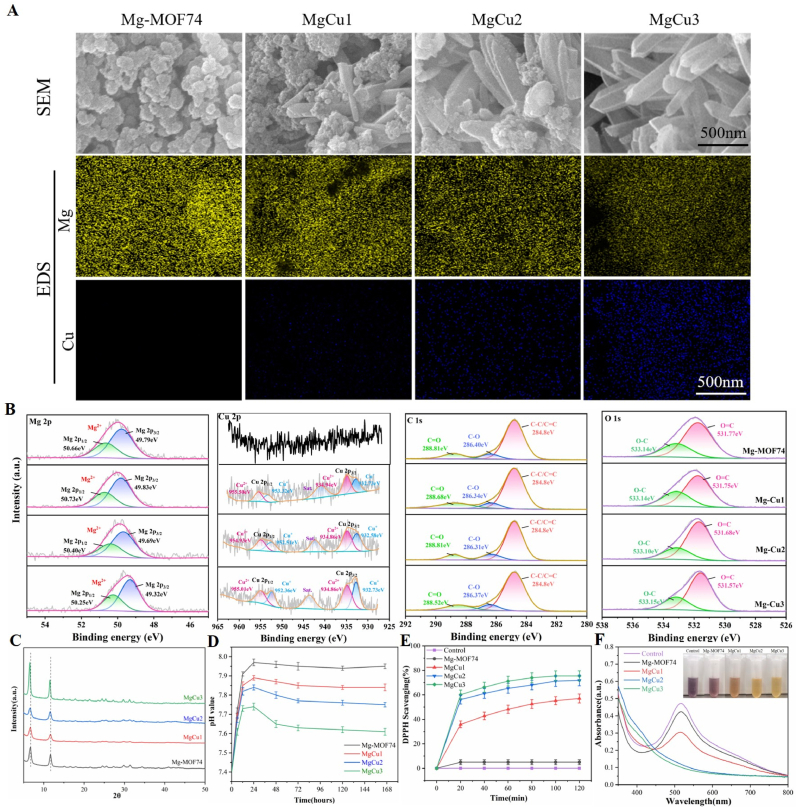
Synthesis and characterization of MgCu-MOF74. (A) Morphologies and EDS mapping images of different MgCu-MOF74 samples. (B) XPS high-resolution spectra of Mg 2p, Cu 2p, C 1s and O 1s. (C) XRD patterns of different samples. (D) Variation trend of the pH value in physiological (pH 7.4) microenvironment. (E) The degradation percentage of DPPH. (F) UV/vis spectra and photograph (inset) of DPPH solution after degradation by different treatments for 120 min.

In this study, Mg-MOF74 doped with varying proportions of Cu was synthesized. As shown in Fig. 2A, pure Mg-MOF74 exhibited a disc-like morphology, while increasing Cu content transformed the crystal structure to a rod-like shape. This change is attributed to the longer Cu-O bond compared to Mg–O, which reduced the electrostatic interaction between Cu and the surrounding oxygen, decreasing the local positive charge of Cu^2+^ [33]. Consequently, Jahn-Teller distortion occurred during the coordination of MgCu-MOF74, leading to the observed morphological change [48]. Energy-dispersive spectroscopy (EDS) analysis confirmed a consistent distribution of Mg, Cu, O, and C elements in the powders (Fig. 2A and S2). The Cu concentration steadily increased while the Mg content decreased from MgCu1 to MgCu3, indicating successful incorporation of Mg and Cu into the MOF-74 structure as a bimetallic framework. Additionally, with increasing Cu content, the particle size of the MOF increased from 161.6 nm (Mg-MOF74) to 282.7 nm (MgCu3) (Fig. S1).

High-resolution XPS spectra (Fig. 2B) revealed that Mg 2p peaks at 49.7 eV and 50.6 eV indicated Mg^2+^ oxidation state. In the Cu 2p spectrum, the peaks located at 932.7 and 952.3 eV were attributed to Cu 2p_3_/_2_ and Cu 2p_1_/_2_ of Cu^+^. Additionally, two peaks corresponding to Cu^2+^ 2p_3_/_2_ (934.9 eV) and Cu^2+^ 2p_1_/_2_ (955 eV) were also exhibited. The data suggest that Cu exists in mixed valence states (Cu^+^ and Cu^2+^) within the samples, implying potential antioxidant functions via oxidation state conversion. In the C 1s spectrum, three fitted peaks at 284.8 eV, 286.4 eV, and 288.8 eV correspond to C-C/C=C, C-O, and C=O bonds, respectively. In the O 1s spectrum, peaks at 531.7 eV and 533.1 eV are attributed to C=O and C-O bonds in the organic ligand structure. XRD patterns (Fig. 2C) showed characteristic MOF-74 peaks, with 6.8° and 11.9° peaks shifting to lower angles due to the larger ionic radius of Cu^2+^ (0.073 nm) compared to Mg^2+^ (0.072 nm), increasing interplanar distance.

To investigate sample stability and ion release behavior, powders were immersed in PBS, and the release of Mg^2+^ and Cu^2+^ was tracked over time. Mg-MOF74 exhibited a burst release of Mg^2+^ within 24 h, followed by negligible further release, suggesting near-complete degradation. For Cu-doped MgCu-MOF74, a slower and sustained release of both Mg^2+^ and Cu^2+^ occurred over 1–5 days (Fig. S3). Typically, MOF materials, held together by weak coordination bonds between ligands and metal ions, degrade easily in physiological environments [49], and rapid metal ion release upon decomposition compromises biocompatibility. To address this, we increased the Cu^2+^ concentration, enhancing MOF-74 stability. This improvement is attributed to structural defects introduced by the heterogeneous metal sites, which strengthen the stability of the terminal M − O bonds [50]. Moreover, released Mg^2+^ ions increased pH in all groups, creating a mildly alkaline microenvironment conducive to bone repair (Fig. 2D).

### 2.3 Antioxidant capabilities of MgCu-MOF74

The antioxidant properties of MgCu-MOF74 were first evaluated using a 1,1-diphenyl-2-picrylhydrazyl (DPPH) free radical scavenging assay. MgCu2 and MgCu3 showed higher DPPH scavenging rates than MgCu1 within 120 min (Fig. 2E), evidenced by pronounced color lightning of DPPH solutions (Fig. 2F), while Mg-MOF74 had no significant effect. To further assess cellular antioxidant defenses, an oxidative stress microenvironment was simulated using H_2_O_2_. MgCu2 and MgCu3 groups exhibited the lowest ROS levels, followed by MgCu1, Mg-MOF74, and the blank group (Fig. S4a). Fluorescence intensity quantification (Fig. S4b) confirmed significant inter-group differences.

The MgCu-MOF74 synthesized in this study effectively scavenges ROS, attributed to the rapid redox cycling between Cu^2+^ and Cu ^+^ oxidation states. Notably, although Mg-MOF74 showed no DPPH scavenging activity in vitro (Fig. 2E and F), it significantly reduced intracellular ROS levels compared to the blank group (Fig. S4). This may be due to the released Mg^2+^, as research indicates that Mg^2+^ can modulate immune responses by regulating cytokine release [51], and enhance antioxidant defenses by upregulating nicotinamide adenine dinucleotide phosphate (NADPH), glutathione, catalase (CAT), and superoxide dismutase (SOD) activities [52].

### 2.4 Effects of MgCu-MOF74 on BMSCs proliferation and osteogenesis

The potential toxicity risks of Metal-Organic Framework (MOF) materials, which consist of metal ions and organic ligands, especially heavy metals, remain a significant concern [43]. There was no significant difference in cell proliferation among the groups after 1 day of culture (Fig. S5a and S5b). However, after 3 days, the MgCu3 group showed a reduced cell survival rate due to the high local release of Cu ions, while the survival rates in the other MOF groups increased, with a more pronounced effect by day 7. Due to the increased cytotoxicity of MgCu3, the Mg-MOF74, MgCu1, and MgCu2 groups were selected for further studies on osteogenic differentiation and angiogenesis. These data delineate the in vitro safe release range of Cu^2+^ as approximately 0.2–0.45 mg/L, whereas concentrations exceeding ∼0.6 mg/L are likely to induce cytotoxicity. The effects of different MOFs on early osteogenic differentiation of BMSCs were evaluated using alkaline phosphatase (ALP) staining, while osteogenic mineralization was assessed via Alizarin Red S staining. As shown in Fig. S5c–f, the MOF-treated groups exhibited deeper ALP staining and larger calcified nodules compared to the blank control group, although no significant differences were observed among the MOF-treated groups. RT-qPCR analysis of osteogenesis-related genes (Fig. S6) revealed significant upregulation of Runx2, Col-I, OCN, and ALP in the MOF-treated groups. Mg, the fourth most abundant cation in the human body, has been shown to positively affect osteogenesis [32]. Cu is essential for bone metabolism. Cu^2+^ mediates angiogenesis and osteogenesis during bone regeneration via the HIF-1 pathway, accelerating vascular infiltration into bone defects and promoting bone repair [53]. The collective findings from these experiments demonstrate that Mg-MOF74, MgCu1, and MgCu2 exhibit excellent biocompatibility and effectively promote the proliferation and osteogenic differentiation of BMSCs.

### 2.5 Angiogenic properties of MgCu-MOF74

Live/dead staining showed that all MOF-treated groups significantly enhanced HUVECs proliferation compared to the control at all time points (Fig. S7a and S7b), with angiogenic effects increasing with Cu content. Subsequently, the migration and tube formation abilities of HUVECs were evaluated. As depicted in Fig. S7c and S7d, HUVECs treated with MOFs exhibited enhanced migration compared to the control group, with wound closure rates positively correlating with Cu content. Furthermore, MOF-treated HUVECs demonstrated improved tube formation, with increased numbers of branches and total tube length (Fig. S7e–g). RT-qPCR analysis of angiogenesis-related genes revealed that MOF-treated HUVECs expressed significantly higher levels of CD31 and VEGF (Fig. S7h). Collectively, these findings highlight the strong angiogenic properties of the synthesized MOFs, with MgCu2 exhibiting the most prominent angiogenic potential. Cu^2+^ is essential for enhancing angiogenic activity by activating signaling pathways involved in endothelial cell migration, differentiation, and proliferation. Notably, the comparison between pure Mg-MOF74 and the control group emphasizes the pro-angiogenic effect of Mg ions (Fig. S7). Studies have shown that Mg^2+^ can enhance HUVEC migration and tubule formation while upregulating markers associated with H-type vessels [15].

Overall, MgCu-MOF74 with varying Cu contents demonstrated excellent antioxidant, osteogenic, and angiogenic properties. Based on these results, MgCu2 was selected as the optimal active particle. We then developed a multifunctional composite scaffold by 3D-printing biodegradable materials with these nanoparticles.

### 2.6 Fabrication and characterization of 3D-printed porous composite scaffolds

**Fig. 3:**
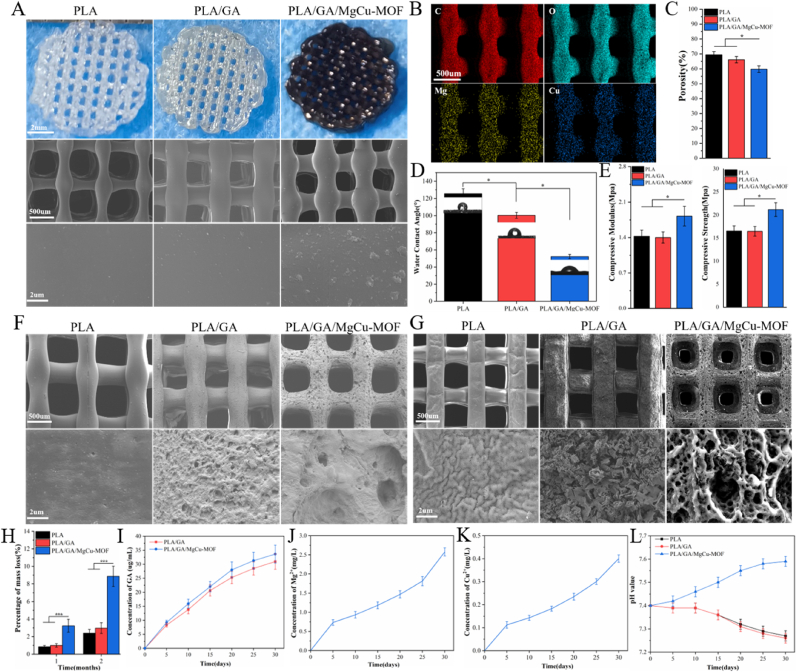
Characterization and degradation behavior of 3D-Printed porous composite scaffolds. (A) Macromorphologies and representative SEM images of different scaffolds. (B) EDS mapping images of PLA/GA/MgCu-MOF. (C) Porosity analyzed by micro-CT. (D) Water contact angles and (E) mechanical properties of different scaffolds. (F, G) SEM images and (H) mass loss of different scaffolds after degradation in pH 7.4 at 37 °C for 30 and 60 days. The released (I) GA, (J) Mg^2+^, (K) Cu^2+^, and (L) the variation trend of the pH value in physiological (pH 7.4) microenvironment. (n = 3; ∗*P* < 0.05, ∗∗*P* < 0.01, ∗∗∗*P* < 0.001).

The macroscopic and microscopic morphologies of different 3D-printed porous scaffold samples are shown in Fig. 3A. PLA scaffolds appeared bright white, PLA/GA scaffolds were pale yellow, and PLA/GA/MgCu-MOF scaffolds turned dark brown due to the addition of MgCu2. SEM revealed that PLA/GA/MgCu-MOF scaffolds had a notably rougher surface than other groups. All scaffolds exhibited uniform, highly interconnected macropores with average sizes of 486 ± 26 μm (PLA), 462 ± 31 μm (PLA/GA), and 419 ± 24 μm (PLA/GA/MgCu-MOF). Scaffolds with pores larger than 300 μm facilitate osteogenesis and cell proliferation by enhancing vascularization for nutrient delivery [54]. EDS analysis confirmed the uniform distribution of Mg and Cu on the surface of PLA/GA/MgCu-MOF scaffolds (Fig. 3B). Mechanical testing showed that the compressive modulus of PLA, PLA/GA, and PLA/GA/MgCu-MOF scaffolds were 1.43 ± 0.12 MPa, 1.40 ± 0.11 MPa, and 1.82 ± 0.19 MPa, respectively (Fig. 3E). The compressive strengths were 16.54 ± 1.12 MPa, 16.48 ± 1.04 MPa, and 21.19 ± 1.49 MPa, respectively (Fig. 3E). A significant difference was observed between the PLA/GA/MgCu-MOF scaffolds and the other two groups (p < 0.05), indicating that the addition of MOF significantly enhanced the mechanical properties of the composite scaffolds, which are comparable to those of human trabecular bone. Porosity values calculated using micro-CT were 69.42 ± 2.12 % (PLA), 66.12 ± 2.11 % (PLA/GA), and 59.82 ± 2.19 % (PLA/GA/MgCu-MOF) (Fig. 3C). Water contact angle measurements (Fig. 3D) revealed that PLA (125.38 ± 5.93°) and PLA/GA (100.28 ± 3.43°) were hydrophobic, whereas PLA/GA/MgCu-MOF showed moderate hydrophilicity (52.19 ± 2.49°). The improved wettability, attributed to GA and MgCu-MOF addition, correlated with SEM-observed surface roughness that enhances cell adhesion.

### 2.7 In vitro degradation behavior

SEM and weight loss analysis (Fig. 3F–H) characterized the degradation of scaffolds in PBS (pH 7.4). After 1 month, both PLA and PLA/GA scaffolds exhibited mild surface erosion characterized by shallow pits and initial pore formation. After 2 months, erosion in PLA and PLA/GA became more pronounced, with deeper pits and increased roughness, yet their overall frameworks remained intact, indicating slow degradation rates and good mechanical stability. In contrast, the PLA/GA/MgCu-MOF scaffold underwent substantial microstructural changes after 1 month, developing interconnected pores, irregular cracks, and rough layered textures that signaled accelerated surface decomposition. By the second month, pore size and connectivity increased, creating an open porous network with fewer bridging regions. Surface roughness intensified, and erosion marks along the edges became more prominent, reflecting an accelerated degradation rate. Despite these changes, the overall framework of PLA/GA/MgCu-MOF remained intact without evident collapse. Weight loss measurements further revealed that after 2 months, the PLA/GA/MgCu-MOF scaffold experienced a mass loss of 8.87 ± 1.15 %, which was significantly higher than that of PLA (2.39 ± 0.44 %) and PLA/GA (2.97 ± 0.62 %). The rapid degradation of PLA/GA/MgCu-MOF may be attributed to the exposure of more loose internal structures after nanoparticle release. Initially, the release rate of GA was fast, gradually slowing down, while the release of Mg^2+^ and Cu^2+^ gradually accelerated due to the water stability of the MgCu-MOF (Fig. 3I–K); after 30 days, cumulative releases reached 33.62 ± 3.2 μg/mL for GA, 2.58 ± 0.11 mg/L for Mg^2+^, and 0.40 ± 0.02 mg/L for Cu^2+^. Notably, degradation of the PLA/GA/MgCu-MOF scaffold increased the solution pH to 7.59 after 30 days, while the pH in the PLA and PLA/GA groups decreased to around 7.27 (Fig. 3L).

### 2.8 In-vitro biocompatibility of PLA/GA/MgCu-MOF

The in vitro performance of the 3D-printed composite scaffolds was evaluated by assessing cell adhesion and proliferation. Live/dead staining (Fig. S8a) after 3 days revealed that all scaffolds supported viable cell adhesion according to their surface morphology, with the PLA/GA/MgCu-MOF group exhibiting a notably higher density of adherent cells. After 7 days, cell numbers increased across all scaffolds; however, the PLA/GA/MgCu-MOF scaffolds hosted densely packed, long, spindle-shaped live cells forming a compact network, with minimal dead cells detected. CCK-8 assays (Fig. S8c) confirmed that cell proliferation on the PLA/GA/MgCu-MOF scaffolds was significantly higher than that on the other groups. To further investigate cellular extensions on the scaffolds, cytoskeleton staining was performed on day 4. As shown in Fig. S8b, BMSCs in all groups displayed typical spindle-shaped structures with intact cytoskeletons. Compared to the sparse cell distribution in the control group, cells on PLA/GA/MgCu-MOF scaffolds exhibited enhanced elongation, larger lamellipodia, and closer intercellular connections. These findings demonstrate that PLA/GA/MgCu-MOF scaffolds provide a favorable microenvironment for cell adhesion and growth, indicating excellent biocompatibility.

### 2.9 In vitro study on the anti-inflammatory effects of PLA/GA/MgCu-MOF

**Fig. 4:**
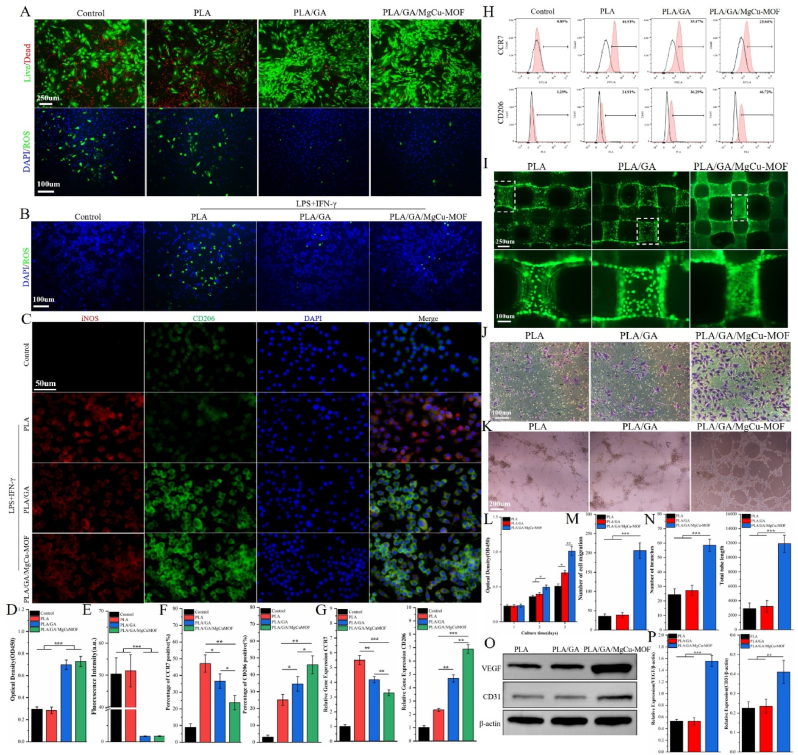
Anti-inflammatory and angiogenic effects of PLA/GA/MgCu-MOF. (A) Live/dead staining (green/red) and fluorescence images of Hoechst 33,342 (blue) and DCFH-DA (green) in BMSCs after 24 h with 100 μM H_2_O_2_. (B) Fluorescence images of RAW264.7 cells stained with Hoechst 33,342 (blue) and DCFH-DA (green) under various treatments. (C) Immunofluorescent staining of iNOS and CD206 in RAW264.7 cells after 7 days of culture with different scaffolds. (D) Absorbance (450 nm) of BMSCs after 24 h under 100 μM H_2_O_2_. (E) ROS fluorescence intensity in BMSCs. (F) Percentage of CCR7 and CD206 positive cells. (G) RT-qPCR analysis of CCR7 and CD206 expression. (H) Flow cytometry of CCR7 and CD206 in RAW264.7 cells. (I) Live/dead staining of HUVECs after 3-day treatment with scaffolds. (J) Crystal violet staining of HUVECs migration with different scaffolds. (K) Tube formation assay for HUVECs cultured with various scaffolds. (L) Absorbance (450 nm) of HUVECs cultured with various scaffolds for 1, 2 and 3 days. (M) Quantification of HUVEC migration. (N) Quantification of the number of branches and total tube length. (O) Western blot analysis and (P) quantification of CD31 and VEGF protein expression in HUVECs treated with different scaffolds. (n = 3; ∗*P* < 0.05, ∗∗*P* < 0.01, ∗∗∗*P* < 0.001).

As shown in Fig. S9, both the PLA/GA and PLA/GA/MgCu-MOF scaffolds achieved approximately 80 % scavenging efficiency within 120 min under dark conditions, while the pure PLA scaffold exhibited negligible activity. After 24 h of H_2_O_2_ exposure, BMSCs cultured with the PLA/GA and PLA/GA/MgCu-MOF scaffolds displayed significantly reduced cell death and lower intracellular ROS levels, as evidenced by live/dead staining and DCFH-DA assays (Fig. 4A, D, and 4E).

An inflammatory stimulation model using RAW264.7 cells was established to assess the scaffolds' immunomodulatory effects. The PLA/GA and PLA/GA/MgCu-MOF scaffolds exhibited the lowest intracellular ROS production, as indicated by reduced green fluorescence (Fig. 4B). Following 24 h of co-culture under inflammatory activation, the PLA/GA and PLA/GA/MgCu-MOF scaffolds demonstrated lower expression of the M1 macrophage marker iNOS and higher expression of the M2 marker CD206 (Fig. 4C and F). Flow cytometry analysis further quantified these differences: the PLA/GA/MgCu-MOF group had a significantly lower proportion of CCR7-positive (M1) macrophages (25.64 %) compared with the PLA (46.93 %) and PLA/GA (35.17 %) groups, while the percentage of CD206-positive (M2) macrophages increased from 24.91 % in the PLA group to 46.72 % in the PLA/GA/MgCu-MOF group (Fig. 4H). This shift in macrophage polarization was corroborated by gene expression analysis showing downregulation of CCR7 and upregulation of CD206 in the PLA/GA/MgCu-MOF group (Fig. 4G). Furthermore, ELISA measurements revealed that macrophages cultured with the PLA/GA/MgCu-MOF scaffold secreted the highest levels of anti-inflammatory cytokines IL-4 and IL-10, while exhibiting the lowest levels of pro-inflammatory cytokines TNF-α and IL-6 (Fig. S10). In line with previous reports highlighting GA's anti-inflammatory properties [36], this study shows that GA-containing scaffolds modulate macrophage polarization and alleviate inflammation via ROS scavenging. Notably, the superior anti-inflammatory efficacy of PLA/GA/MgCu-MOF compared to PLA/GA can be attributed to the antioxidant capacity of MgCu-MOF.

Collectively, these results demonstrate that both PLA/GA and PLA/GA/MgCu-MOF scaffolds enhance anti-inflammatory cytokine secretion while suppressing pro-inflammatory cytokines, effectively promoting M1-to-M2 macrophage polarization. Notably, PLA/GA/MgCu-MOF exhibits more potent and efficient immunomodulatory activity.

### 2.10 In vitro study on the angiogenesis of PLA/GA/MgCu-MOF

The proliferation of HUVECs cultured on scaffolds for 3 days was evaluated using live/dead staining (Fig. 4I). The results showed that the surface of PLA/GA/MgCu-MOF scaffolds supported a significantly higher number of HUVECs compared to the PLA/GA and PLA groups. Although initial CCK-8 assays on day 1 were similar across all groups, marked increases in OD values were observed for the PLA/GA/MgCu-MOF group by days 2 and 3 (Fig. 4L). Cell migration was evaluated using crystal violet staining coupled with quantitative analysis (Fig. 4J and M), which revealed superior migratory capacity on the PLA/GA/MgCu-MOF scaffolds relative to the other groups. Tube formation assays revealed similar trends, with HUVECs on PLA/GA/MgCu-MOF scaffolds forming well-defined tubular structures within 6 h, while cells in the PLA/GA and PLA groups primarily formed numerous vascular branches with discontinuous walls (Fig. 4K). Statistical comparisons further confirmed that both the number of branches and total tube length were significantly greater in the PLA/GA/MgCu-MOF group (Fig. 4N). Western blot analyses performed after 48 h showed that the PLA/GA/MgCu-MOF scaffolds induced the highest expression levels of angiogenic markers VEGF and CD31, as validated by grayscale quantification (Fig. 4O and P). These collective findings highlight the excellent angiogenic properties of the PLA/GA/MgCu-MOF scaffold.

### 2.11 In vitro study on the neurogenesis of PLA/GA/MgCu-MOF

**Fig. 5:**
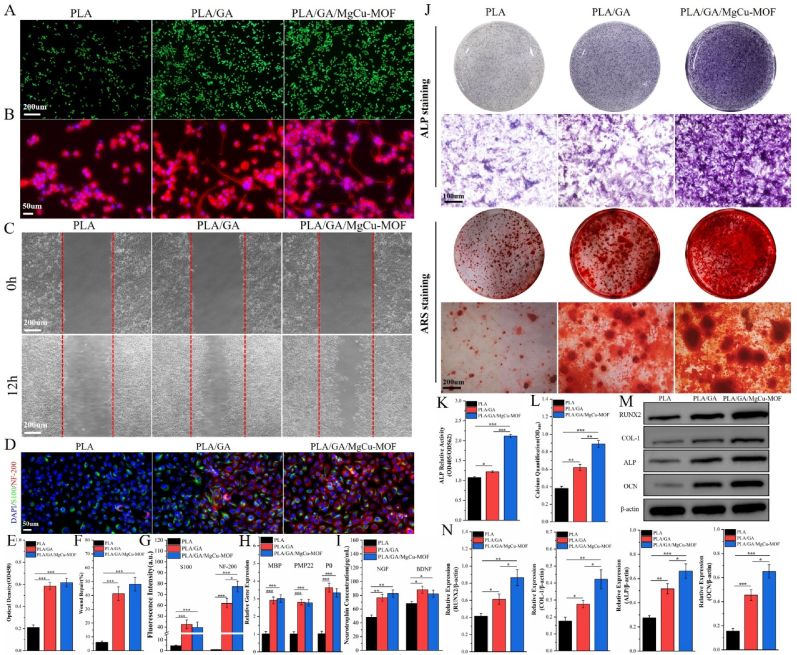
Neurogenesis and osteogenic differentiation of PLA/GA/MgCu-MOF. (A) Live/dead staining of RSC-96 after 2-day treatment with scaffolds. (B) Fluorescence images of cytoskeleton (red, phalloidin) and nuclei (blue, DAPI) in RSC-96 cells. (C) Wound healing assay of RSC-96 after different treatments. (D) Immunofluorescence staining of RSC-96 with NF-200 (red), S100 (green), and DAPI (blue) cultured with different scaffolds. (E) Absorbance (450 nm) of RSC-96 cultured with various scaffolds for 2 days. (F) Wound repair ratio. (G) Fluorescence intensity of NF-200 and S100 in BMSCs. (H) RT-qPCR analysis of MBP, PMP22 and P0 expression. (I) The neurotrophin secretion of NGF and BDNF of RSC-96 cultured with different scaffolds. (J) Alkaline phosphatase (ALP) staining on 7 days and Alizarin red staining on 14days. (K, L) Quantitative analysis of ALP and Alizarin red staining results. (M) Western blot analysis and (N) quantification of Runx2, Col-I, OCN, and ALP protein expression in BMSCs treated with different scaffolds. (n = 3; ∗*P* < 0.05, ∗∗*P* < 0.01, ∗∗∗*P* < 0.001).

Building on the GA-induced neurogenic effects noted in Fig. 1, we assessed the impact of PLA/GA/MgCu-MOF scaffolds on RSC-96 cell behavior. After 2 days of culture, live/dead staining combined and CCK-8 assays demonstrated that both the PLA/GA and PLA/GA/MgCu-MOF groups supported a significantly higher cell number than the pure PLA scaffold (Fig. 5A and E). Cytoskeletal imaging revealed that while cells on the PLA scaffold remained largely rounded, those on the composite scaffolds transitioned to a spindle-like morphology with elongated axonal projections and enhanced intercellular connectivity (Fig. 5B), indicating that composite scaffolds not only facilitate cell adhesion but also promote cellular maturation. Moreover, in a scratch assay performed over 12 h, the composite scaffold groups exhibited a greater number of cells migrating along the wound edge than the PLA group, thereby substantially increasing the rate of wound repair (Fig. 5C and F). The observation indicates that GA released from the composite scaffolds accelerates RSC-96 cell migration— critical for axonal guidance and subsequent myelination. Immunofluorescence staining further confirmed significantly elevated expression of S100 and NF-200 in composite scaffold groups relative to PLA (Fig. 5D and G), with RT-qPCR analysis demonstrating pronounced upregulation of myelin-associated genes (MBP, PMP22, P0) in corresponding cell cultures (Fig. 5H). Given RSC-96 cells' role in modulating the neural microenvironment via neurotrophic factor secretion, ELISA assays quantified NGF and BDNF levels. Notably, cells cultured on PLA/GA/MgCu-MOF scaffolds secreted significantly higher concentrations of these factors than other groups (Fig. 5I). Collectively, these findings establish that the PLA/GA/MgCu-MOF scaffold not only promotes morphological maturation of RSC-96 cells but also enhances their neurotrophic factor secretory capacity, thereby creating an optimized neuroregenerative microenvironment.

### 2.12 In vitro study on the osteogenic differentiation of PLA/GA/MgCu-MOF

Early osteogenic differentiation was assessed via ALP staining, and mineralization was evaluated using Alizarin Red S. After 7 days of co-culture, BMSCs in the PLA/GA/MgCu-MOF group exhibited notably deeper ALP staining compared to the PLA/GA and PLA groups (Fig. 5J). After 14 days, the PLA/GA/MgCu-MOF group also demonstrated more extensive and larger calcium nodule formation, as observed both microscopically and on the culture plates (Fig. 5J). Quantitative analysis confirmed that both ALP activity and calcium deposition were significantly higher in the PLA/GA/MgCu-MOF group relative to the other groups (Fig. 5K and L). To further elucidate the osteogenic effects, the expression levels of key osteogenic genes—Runx2, Col-I, OCN, and ALP—were measured at 7 and 14 days. The results revealed that the PLA/GA/MgCu-MOF scaffolds induced a marked upregulation of these markers compared to the PLA/GA group, with both composite scaffold groups outperforming the PLA group (Fig. S11). Consistently, grayscale analysis of osteogenic protein expression (Runx2, Col-I, Ocn, and ALP) in BMSCs showed significantly higher levels in the PLA/GA/MgCu-MOF group (Fig. 5M and N). Collectively, these findings indicate that the incorporation of osteogenic MgCu-MOF into the PLA/GA/MgCu-MOF scaffold effectively enhances the osteogenic differentiation of BMSCs.

### 2.13 Osteogenic mechanism of PLA/GA/MgCu-MOF

**Fig. 6:**
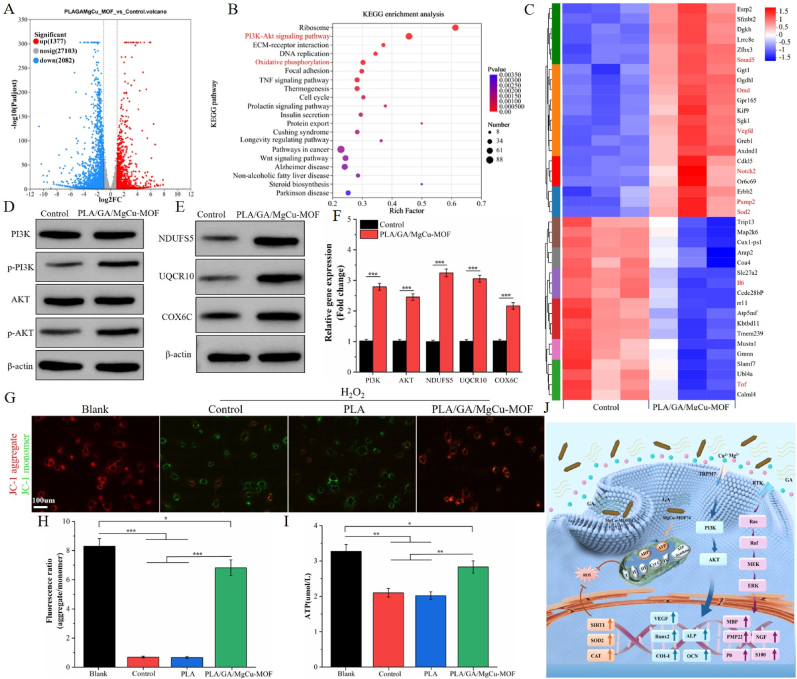
Osteogenic mechanism of PLA/GA/MgCu-MOF. (A) Volcano plot of the DEGs between PLA/GA/MgCu-MOF and Control groups. (B) KEGG enrichment analysis and (C) A heatmap of DEGs. (D, E) Western blot analysis and (F) quantification of PI3K, p-PI3K, AKT, p-AKT, NDUFS5, UQCR10, and COX6C. (G) JC-1 fluorescence staining of BMSCs treated under various conditions. (H) Ratio of red to green fluorescence intensity in JC-1 staining. (I) ATP levels in BMSCs treated with different conditions. (J) Schematic diagram showing the mechanism by which PLA/GA/MgCu-MOF promotes osteogenesis. (n = 3; ∗*P* < 0.05, ∗∗*P* < 0.01, ∗∗∗*P* < 0.001).

To investigate the regulatory mechanisms of PLA/GA/MgCu-MOF scaffolds on osteogenic differentiation, BMSCs were co-cultured with the scaffolds for 14 days followed by transcriptomic sequencing. The volcano plot revealed 1377 upregulated and 2082 downregulated genes (Fig. 6A). KEGG enrichment analysis highlighted significant enrichment in the oxidative phosphorylation and PI3K-Akt signaling pathway (Fig. 6B). GO analysis further revealed enriched biological processes (ossification, osteoblast differentiation, angiogenesis, immune regulation, bone mineralization), cellular components (microtubules), and molecular functions (metal ion binding, tubulin binding, microtubule motor activity), with key terms marked in red (Fig. S12). Compared to the control, PLA/GA/MgCu-MOF significantly upregulated genes related to osteogenic differentiation, angiogenesis, neurogenesis, and anti-inflammatory responses, including Smad5, Omd, VEGF, Notch2, PXMP2, and SOD2 (Fig. 6C, marked in red). Smad5 (BMP pathway effector) promotes osteoblast differentiation and bone matrix mineralization while inhibiting resorption [55]. Omd (mineralized tissue proteoglycan) regulates cell adhesion and bone mineralization [56]. VEGF is crucial for endothelial cell proliferation, migration, and angiogenesis [57]. Notch2, a subtype of the Notch receptor, regulates the proliferation and differentiation of neural progenitor cells and participates in the repair process after neural injury [47]. PXMP2 supports peroxisomal membrane function, promotes hydrogen peroxide degradation, and protects cells from oxidative stress [58]. SOD2, an important antioxidant enzyme, protects mitochondria and other cell structures from oxidative damage [59]. Furthermore, PLA/GA/MgCu-MOF markedly reduced the expression of pro-inflammatory cytokines IL-6 and TNF (Fig. 6C, marked in red).

To validate these findings, we assessed the expression of key proteins in the PI3K-Akt and oxidative phosphorylation signaling pathways. Compared to the control, cells treated with PLA/GA/MgCu-MOF showed significantly increased phosphorylation of PI3K and AKT, along with upregulation of key oxidative phosphorylation proteins NDUFS5, UQCR10, and COX6C (Fig. 6D–F). The PI3K-Akt signaling pathway regulates cell proliferation, migration, metabolism, and immune response [60], while Mg^2+^ and Cu^2+^ play key roles in regulating the PI3K-Akt and HIF-1 signaling pathways associated with angiogenesis [61]. Specifically, Mg^2+^ stimulates angiogenesis via transient receptor potential (TRPM7) channel activation [62], and PI3K-Akt upregulates VEGF, OPG, Runx2, and ALP for osteogenesis [7,60]. Oxidative phosphorylation is the main process of cellular energy production, occurring on the inner mitochondrial membrane, playing a crucial role in regulating cellular redox reactions and energy metabolism. Given that ROS-mediated oxidative stress can lead to mitochondrial dysfunction and insufficient energy supply, we assessed the impact of PLA/GA/MgCu-MOF on mitochondrial function by measuring ATP production and mitochondrial membrane potential under oxidative stress. JC-1 staining showed that H_2_O_2_ reduced mitochondrial membrane potential (decreased red/green fluorescence ratio), whereas PLA/GA/MgCu-MOF significantly restored this ratio (Fig. 6G and H). ATP assays confirmed enhanced bioenergetics in the scaffold group (Fig. 6I). These results suggest that PLA/GA/MgCu-MOF improves mitochondrial dysfunction caused by oxidative stress. Furthermore, antioxidant gene expression (Fig. S13) revealed upregulation of SIRT1, SOD2, and CAT, reducing intracellular ROS.

In conclusion, PLA/GA/MgCu-MOF scaffolds promote osteogenic differentiation and angiogenesis by activating the PI3K-Akt pathway, enhancing oxidative phosphorylation, upregulating antioxidant genes, and restoring mitochondrial bioenergetics under oxidative stress (Fig. 6J).

### 2.14 In vitro study on the regulation of intercellular crosstalk by PLA/GA/MgCu-MOF

**Fig. 7:**
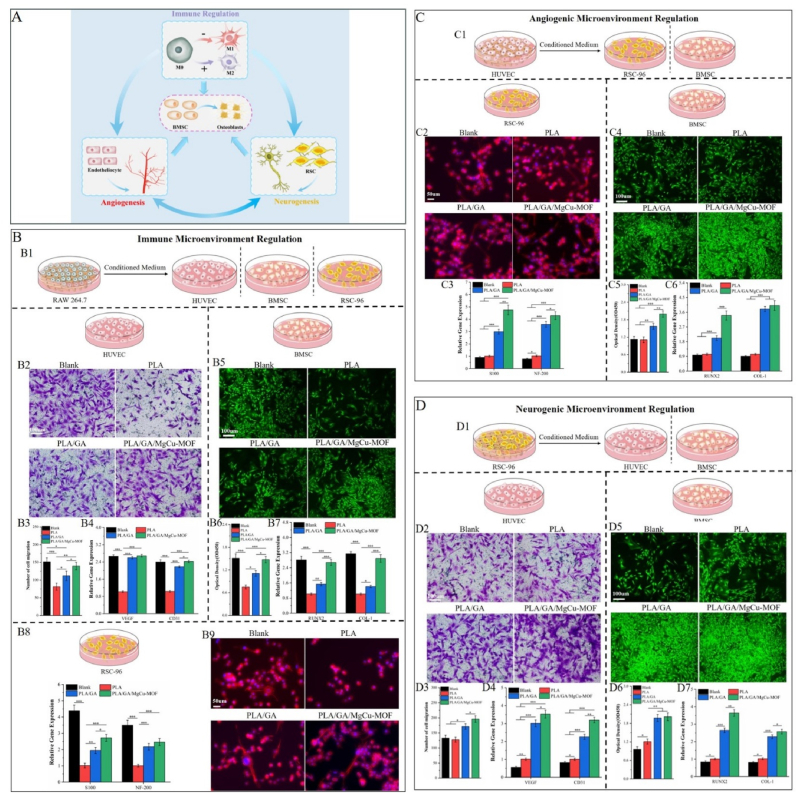
Regulation of intercellular crosstalk by PLA/GA/MgCu-MOF. (A) Schematic diagram of intercellular crosstalk. (B1) Schematic diagram of HUVECs, BMSCs, and RSC-96 in the conditioned medium from RAW 264.7 treated with various scaffolds. (B2) Crystal violet staining, (B3) quantification and (B4) relative gene expression of HUVECs. (B5) Live/dead staining, (B6) quantification and (B7) relative gene expression of BMSCs. (B8) Relative gene expression and (B9) cytoskeleton staining of RSC-96 in the conditioned medium. (C1) Schematic diagram of RSC-96 and BMSCs in the conditioned medium from HUVECs treated with various scaffolds. (C2) Cytoskeleton staining and (C3) relative gene expression of RSC-96. (C4) Live/dead staining, (C5) quantification and (C6) relative gene expression of BMSCs in the conditioned medium. (D1) Schematic diagram of HUVECs and BMSCs in the conditioned medium from RSC-96 treated with various scaffolds. (D2) Crystal violet staining, (D3) quantification and (D4) relative gene expression of HUVECs. (D4) Live/dead staining, (D5) quantification and (D6) relative gene expression of BMSCs in the conditioned medium. (n = 3; ∗*P* < 0.05, ∗∗*P* < 0.01, ∗∗∗*P* < 0.001).

The microenvironmental damage in glucocorticoid-induced osteonecrosis stems from the imbalance of multiple cell types and their intricate interactions. Merely regulating single-cell behavior is insufficient to restore this microenvironment. Intercellular crosstalk—mediated primarily by cell junctions and paracrine signaling—plays a pivotal role in tissue regeneration [63]. Therefore, this study adopts an indirect co-culture model to evaluate the ability of PLA/GA/MgCu-MOF scaffolds to improve the microenvironment by regulating multicellular crosstalk. RAW 264.7, HUVECs, or RSC-96 cells were cultured on different scaffolds, and the supernatants were collected and used to culture other cell types to study the effects of different scaffolds on promoting cell-to-cell crosstalk (Fig. 7A). The blank group represents natural cell behavior without intercellular interactions (Fig. 7).

We first assessed the behavior of HUVECs, BMSCs, and RSC-96 cells in the conditioned medium from macrophages treated with various scaffolds (Fig. 7B1). The results showed that PLA/GA/MgCu-MOF treatment significantly increased HUVECs proliferation, tube formation, and migration (Fig. S14a, 7B2 and 7B3), accompanied by upregulated VEGF and CD31 gene expression (Fig. 7B4). Similarly, BMSCs cultured with this scaffold's conditioned media showed increased cell counts (Fig. 7B5 and 7B6) and upregulated Runx2 and Col-I expression (Fig. 7B7). Moreover, RSC-96 cells also exhibited enhanced proliferation (Fig. S14b), upregulated S100 and NF-200 expression (Fig. 7B8), and prominent axon elongation with tighter intercellular connections (Fig. 7B9). These findings suggest that PLA/GA/MgCu-MOF scaffold optimizes the immune microenvironment by promoting regenerative crosstalk among macrophages, HUVECs, BMSCs, and RSC-96 cells, enhancing angiogenesis, myelination, and osteogenesis.

Next, we co-cultured RSC-96 cells and BMSCs with HUVECs treated with various scaffolds to examine the angiogenic microenvironment (Fig. 7C1). PLA/GA/MgCu-MOF-treated HUVECs significantly promoted RSC-96 proliferation (Fig. S15), upregulated S100 and NF-200 gene expression (Fig. 7C3), and promoted cytoskeletal reorganization and axon elongation (Fig. 7C2), indicating improved neuronal maturation. For BMSCs, PLA/GA/MgCu-MOF-treated HUVECs promoted cell proliferation and upregulated the gene expression of Runx2 and Col-I (Fig. 7C4-6), demonstrating that the scaffold optimizes the angiogenic microenvironment to facilitate RSC-96 cells maturation and BMSCs osteogenic differentiation. This effect is likely due to enhanced cytokine and growth factor secretion by the treated HUVECs.

Furthermore, we studied the neurogenic microenvironment by co-culturing HUVECs and BMSCs with differently treated RSC-96 cells (Fig. 7D1). PLA/GA/MgCu-MOF-treated RSC-96 cells significantly promoted HUVECs proliferation, angiogenesis, and migration (Fig. S16, 7D2 and 7D3), with upregulation of VEGF and CD31 gene expression (Fig. 7D4). These findings suggest that RSC-96 cells secretions treated with PLA/GA/MgCu-MOF significantly promote HUVECs angiogenic behavior, highlighting the importance of neurovascular development. Additionally, BMSCs showed increased cell numbers and upregulated osteogenic gene expression (Runx2 and Col-I) in the PLA/GA/MgCu-MOF group compared to other groups (Fig. 7D5-7). These findings emphasize the positive interplay between RSC-96 maturation, HUVEC vascularization, and BMSC osteogenic differentiation, further validating the efficacy of PLA/GA/MgCu-MOF scaffolds in optimizing the neurogenic microenvironment.

In conclusion, our findings demonstrate that PLA/GA/MgCu-MOF scaffold optimizes the microenvironment by releasing various bioactive components such as GA, Mg^2+^, and Cu^2+^, which regulate specific cell behaviors and significantly enhance multicellular crosstalk. These findings indicate that the PLA/GA/MgCu-MOF scaffold can effectively improve immune-angiogenic-neurogenic microenvironments synergistically highlights its potential for treating SAON–related bone defects.

### 2.15 Immunomodulatory, angiogenic, neurogenic, and osteogenic effects of PLA/GA/MgCu-MOF in vivo

**Fig. 8:**
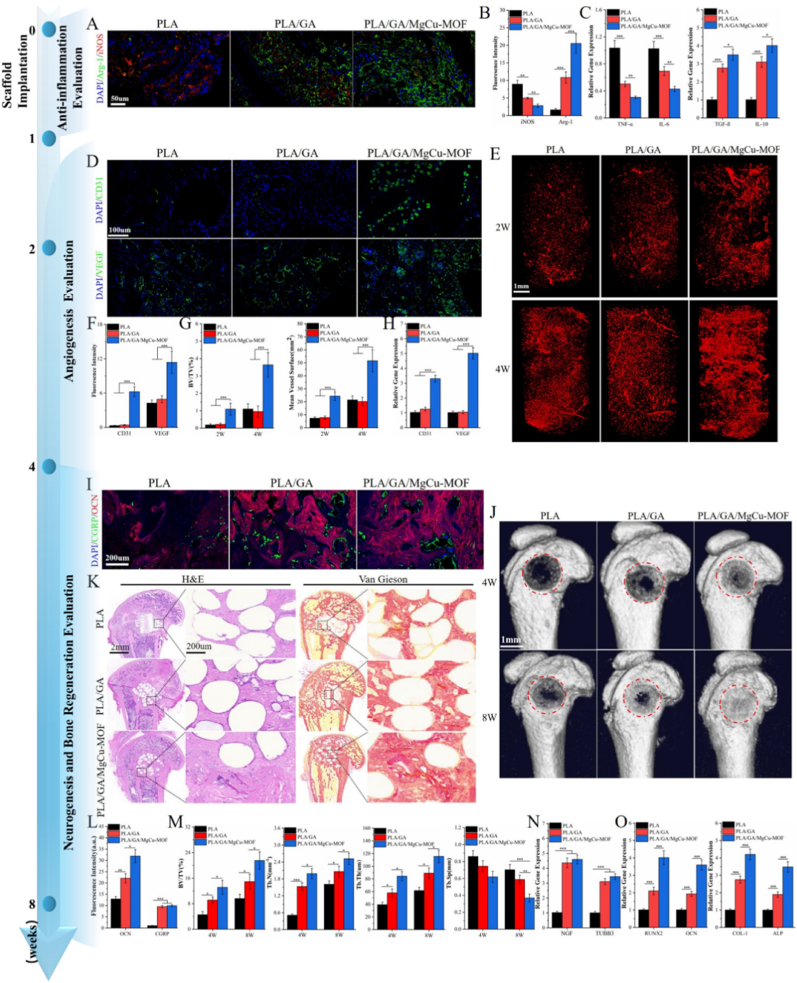
Immunomodulatory, Angiogenic, Neurogenic, and Osteogenic Effects In Vivo. (A) Immunofluorescent staining and (B) quantification of iNOS and Arg1 at 7 days after scaffolds implantation. (C) RT-qPCR analysis of TNF-ɑ, IL-6, TGF-β and IL-10 gene expression. (D) Immunofluorescent staining and (F) quantification of CD31 and VEGF at 21 days after scaffolds implantation. (E) 3D reconstructed images of the internal vessels detected by micro-CT in different scaffolds at 2 and 4 weeks after implantation. (G) Quantitative analysis of mean vessel surface and vessel volume/total volume (BV/TV) inside the scaffolds. (H) RT-qPCR analysis of CD31 and VEGF gene expression. (I) Immunofluorescent staining and (L) quantification of OCN and CGRP at 4 weeks after scaffolds implantation. (J) New bone tissue formation in the scaffolds determined by micro-CT at 4 and 8 weeks after implantation. (K) H&E and Van Gieson staining of bone inside the scaffolds. (M) Quantitative analysis of BV/TV, trabecular number (Tb.N), trabecular thickness (Tb.Th) and trabecular separation (Tb.Sp) at 4 and 8 weeks. (N, O) RT-qPCR analysis of NGF, TUBB3, RUNX2, OCN, Col-I, and ALP gene expression. (n = 3; ∗*P* < 0.05, ∗∗*P* < 0.01, ∗∗∗*P* < 0.001).

The hematoma inflammatory phase plays a crucial role in bone healing. Therefore, we investigated the early immunomodulatory effects of scaffolds during bone repair. Immunofluorescence staining was performed to assess the immune microenvironment 1 week after scaffold implantation in SAON rats, focusing on M1 macrophage (iNOS) and M2 macrophage (Arg-1) markers. As shown in Fig. 8A and B, the PLA/GA/MgCu-MOF group exhibited significantly reduced iNOS expression and enhanced Arg-1 expression compared to PLA and PLA/GA groups. Additionally, qPCR analysis revealed that the PLA/GA/MgCu-MOF group significantly upregulated TGF-β and IL-10 expression, while suppressing IL-6 and TNF-α levels (Fig. 8C). These findings suggest that the PLA/GA/MgCu-MOF group mitigated inflammation by promoting M2 macrophage polarization, thus creating a favorable immune microenvironment conducive to bone repair.

Next, vascularization in bone defect sites was evaluated at multiple time points. 2 weeks after scaffold implantation, the PLA/GA/MgCu-MOF scaffolds exhibited more mature and continuous blood vessels, whereas the vessels in the PLA and PLA/GA groups were fragmented and incomplete, indicative of early-stage angiogenesis. By 4 weeks, the PLA/GA/MgCu-MOF group showed a substantial increase in mature, continuous blood vessels, while the PLA and PLA/GA groups displayed primarily branch-like new vessels with limited maturity and continuity (Fig. 8E). Quantitative analysis demonstrated significantly higher mean vessel surface area and the vessel volume-to-total volume ratio (BV/TV) ratio in the PLA/GA/MgCu-MOF group at both time points (Fig. 8G), with no significant differences between control groups. Immunofluorescence staining of CD31 and VEGF in femoral slices at 4 weeks confirmed these findings, showing substantially higher expression in the PLA/GA/MgCu-MOF group (Fig. 8D and F), consistent with qPCR results (Fig. 8H).

Finally, to assess neurogenesis and bone regeneration, immunofluorescence staining for CGRP and OCN was performed. The PLA/GA/MgCu-MOF group showed enhanced CGRP and OCN fluorescence (Fig. 8I and L), indicating that the scaffold promotes neurogenesis and bone regeneration. Studies have demonstrated that sensory nerve-derived CGRP is a key mediator linking nerve function and bone regeneration, promoting the regeneration of the bone microvascular system and bone formation both in vivo and in vitro [18,24]. Representative 3D reconstructions of new bone ingrowth within the porous PLA scaffolds are shown in Fig. 8J. In all groups, new bone grew from the defect edges towards the scaffold centers. The interconnected porous channels of the scaffolds provided structural support for tissue growth. Over time, more bone ingrowth was observed at 8 weeks compared to 4 weeks in all groups. However, the degree of new bone integration in the PLA and PLA/GA groups was notably lower than in the PLA/GA/MgCu-MOF group. Notably, at both time points, the PLA/GA/MgCu-MOF group exhibited significantly more new bone formation at the defect center, while the PLA and PLA/GA groups showed limited bone growth near the defect edges, with negligible bone tissue observed at the scaffold centers. Microstructural analysis further revealed that bone tissue formation at 8 weeks was greater in all groups compared to 4 weeks. The PLA/GA/MgCu-MOF group demonstrated significantly higher bone mineral density (BMD), bone volume-to-total volume ratio (BV/TV), and trabecular thickness (Tb.Th) at both time points compared to the PLA and PLA/GA groups, with trabecular separation (Tb.Sp) showing the opposite trend (Fig. 8M). Representative histological images at 8 weeks post-implantation are shown in Fig. 8K. In hematoxylin-eosin (HE) stained sections, the PLA/GA/MgCu-MOF group exhibited well-formed bone trabeculae containing red bone marrow and significantly more new blood vessels compared to the PLA and PLA/GA groups. In Van Gieson (V-G) stained sections, red-stained bone tissue was attached to the scaffold edges in the PLA and PLA/GA groups, with limited new bone growth into the scaffold. In contrast, the PLA/GA/MgCu-MOF scaffolds displayed more extensive red-stained new bone growth, with increased growth area and integration as the new bone surrounded and fused with the scaffold. Additionally, qPCR analysis showed that the PLA/GA/MgCu-MOF scaffolds significantly promoted the expression of neurogenesis-related genes (NGF and TUBB3) and osteogenic differentiation-related genes (Runx2, Col-I, OCN, and ALP) (Fig. 8N).

## 3 Conclusion

In summary, based on the pathophysiology of SAON‐related bone defects, we have engineered a multifunctional PLA/GA/MgCu-MOF 3D-printed porous scaffold with excellent mechanical properties, degradability, and biocompatibility. MgCu-MOF74 exhibits antioxidant capacity, controllable release of metal ions, and osteo-angiogenic properties. The incorporation of GA and dual-ion synergy enabled the scaffold to modulate specific cellular behaviors and markedly enhances intercellular crosstalk, thereby optimizing the local multidimensional microenvironment. The scaffold's synergistic immunomodulatory, angiogenic, and neurogenic activities effectively ameliorate the osteonecrotic milieu and promote bone regeneration. Mechanistically, it regulates oxidative phosphorylation, up-regulates antioxidant gene expression to restore mitochondrial function under oxidative stress, and directs macrophage polarization toward an anti-inflammatory phenotype. Moreover, by activating the MAPK/ERK and PI3K-Akt signaling pathways, the scaffold further stimulates neurogenesis, induces neovascularization, and enhances osteogenic differentiation. In vivo assays confirmed that the PLA/GA/MgCu-MOF scaffold substantially accelerates bone repair in SAON‐related defects. Collectively, these findings highlight the therapeutic potential of this composite scaffold for treating SAON‐related bone defects.

## 4 Experimental section

The experimental details are provided in the Supplementary Information.

## CRediT authorship contribution statement

**Yongbo Li:** Writing – original draft, Visualization, Methodology, Investigation, Funding acquisition, Formal analysis, Data curation, Conceptualization. **Yifei Guo:** Software, Methodology, Investigation. **Yuanpei Cheng:** Methodology. **Xiaodong Liu:** Formal analysis. **Hengren Li:** Formal analysis. **Chen Liu:** Validation, Resources. **Xipeng Chen:** Validation, Resources. **Heng Yang:** Conceptualization. **Xingzhi Jing:** Conceptualization. **Xiaoyang Liu:** Validation, Supervision, Funding acquisition. **Han Wu:** Validation, Supervision, Funding acquisition. **Min Guo:** Writing – review & editing, Visualization, Supervision, Resources, Funding acquisition, Formal analysis, Conceptualization. **Peibiao Zhang:** Validation, Supervision, Funding acquisition. **Xingang Cui:** Writing – review & editing, Visualization, Supervision, Resources, Funding acquisition, Formal analysis, Conceptualization.

## Declaration of competing interest

The authors declare that they have no known competing financial interests or personal relationships that could have appeared to influence the work reported in this paper.

## Data Availability

Data will be made available on request.
