# Supplementary material for: Dual-metal-organic framework and gallic acid incorporated 3D-printed scaffolds: Revolutionizing refractory bone defect repair through immune-angiogenic-neurogenic synergy
Source: Mater Today Bio. 2025 Sep 19;35:102323. doi: 10.1016/j.mtbio.2025.102323 (PMC12512983; doi:10.1016/j.mtbio.2025.102323)
Supplement: Multimedia component 1 [file mmc1.docx]

**Dual-Metal-Organic Framework and Gallic Acid Incorporated 3D-Printed Scaffolds: Revolutionizing Refractory Bone Defect Repair through Immune-Angiogenic-Neurogenic Synergy**

Yongbo Li^a^, Yifei Guo^a^, Yuanpei Cheng^b^ ^c^, Xiaodong Liu^a^, Hengren Li^a^, Chen Liu^a^, Xipeng Chen^c^, Heng Yang^a^, Xingzhi Jing^a^, Xiaoyang Liu^a^, Han Wu^c^, Min Guo ^b,^*, Peibiao Zhang^b,^*, Xingang Cui^a,^*

^a^ Department of Spine Surgery, Shandong Provincial Hospital Affiliated to Shandong First Medical University, Jinan, 250000, Shandong, P. R. China

^b^ State Key Laboratory of Polymer Science and Technology, Changchun Institute of Applied Chemistry, Chinese Academy of Sciences, Changchun 130022, P. R. China.

^c^ Department of Orthopedics, China-Japan Union Hospital of Jilin University, Changchun 130000, P. R. China

***Corresponding Author:**

Professor Min Guo, State Key Laboratory of Polymer Science and Technology, Changchun Institute of Applied Chemistry, Chinese Academy of Sciences, Changchun 130022, China; E-mail: guomin@ciac.ac.cn

Professor Peibiao Zhang, State Key Laboratory of Polymer Science and Technology, Changchun Institute of Applied Chemistry, Chinese Academy of Sciences, Changchun 130022, China; E-mail: zhangpb@ciac.ac.cn

Professor Xingang Cui, Department of Spine Surgery, Shandong Provincial Hospital Affiliated to Shandong First Medical University, Jinan, 250000, China; E-mail: [cuixingang@sdfmu.edu.cn](mailto:cuixingang@sdfmu.edu.cn);

**1. Materials and methods**

**1.1 Synthesis of MgCu-MOF74**

A one-step hydrothermal process was used to create MgCu-MOF74. First, a solvent combination comprising 32.25 mL of N, N-dimethylformamide (DMF), 2.5 mL of ethanol, and 2.5 mL of deionized water was used to dissolve 79.2 mg of powdered 2,5-dihydroxyterephthalic acid (DHTA) and 50 mg of polyvinyl pyrrolidone (PVP). Following that, different molar ratios of Cu(NO₃)₂·3H₂O and Mg(NO₃)₂·6H₂O (**Table S1**) were added to the solution while stirring until they were completely dissolved. After being moved into autoclaves, the mixes underwent a 24-hour heat treatment at 130 °C in a muffle furnace, followed by a 6-hour natural cooling period. The resulting precipitates were collected, cleaned three times using ethanol and DMF, and vacuum-dried for 72 hours at 60 °C. Mg-MOF74, MgCu1, MgCu2, and MgCu3 were the names given to the produced powders, which corresponded to the Cu doping levels shown in **Table S1**.

**1.2 Preparation of PLA/GA/MgCu-MOF Scaffolds**

To fabricate composite scaffolds, MgCu-MOF74 (5 wt%) and GA(3%) were homogeneously blended with PLA (Mw = 10 kDa, purchased from Shandong Institute of Medicine Instruments, China) using a twin-screw extruder. The blend was then melted, extruded, and stretched at a controlled rate to achieve the desired filament diameter. Using predetermined parameters, PLA/GA/MgCu-MOF scaffolds were fabricated via 3D printing with specific nozzle temperatures and printing speeds. Pure PLA scaffolds and PLA/GA scaffolds were also prepared for comparative analysis. Scaffold dimensions (20 × 20 × 3 mm and φ8 × 2 mm) were designed using SolidWorks 2017, with printing parameters configured in Simplify3D software (infill density: 50%, printing speed: 10 mm/s).

**1.3 Characterization of MgCu-MOF74 and** **PLA/GA /MgCu-MOF Scaffolds**

The microscopic morphology and elemental distribution of the scaffolds were investigated using energy-dispersive spectroscopy (EDS) and a scanning electron microscope (LIBRA 200 CS, Carl Zeiss Co., Germany). An X-ray diffractometer was used to determine the materials' crystalline structures (XRD; MAX-2250, Japan). An X-ray photoelectron spectrometer (XPS; 250Xi, Thermo Scientific, USA) was used to identify the elements' chemical states. The water contact angle (WCA) of scaffold surfaces was measured by depositing 2 μL of deionised water using a Krüss DSA100 contact-angle analyser.

In order to assess the mechanical characteristics of scaffolds, 10×10×10 mm³ cubes were created. After that, they were sent through a universal testing equipment that was operated by a computer at a displacement rate of 1 mm per minute until they failed. According to ISO 844:2007 guidelines, five samples from each group were examined, and Young's modulus was determined using the load-displacement curves. Scaffold porosity was measured using micro-computed tomography (Micro-CT; μCT 40, SCANCO Medical, Bassersdorf, Switzerland) at 45 kV, 145 μA, and a spatial resolution of 14 μm. CTAn software was used for segmentation analysis and data reconstruction.

**1.4 Release Detection of Mg²⁺, Cu²⁺, and GA**

To investigate ion release, three samples from each group were immersed in 20 millilitres of phosphate-buffered saline (PBS) solution (pH 7.4). The samples were incubated at 37 °C for the designated durations of 5, 10, 15, 20, 25, and 30 days. At each time point, aliquots of the solutions were collected, and the quantities of Mg²⁺ and Cu²⁺ ions were measured using inductively coupled plasma atomic emission spectrometry (ICP-AES).

Scaffolds containing GA were submerged in 20 mL of PBS (pH 7.4) and incubated at 37 °C for the same amount of time in order to analyze GA release. The concentration of GA was measured using a NanoDrop One Microvolume UV-Vis spectrophotometer (Thermo Fisher Scientific, USA) calibrated at 259 nm. A standard calibration curve was used to determine the quantity of released GA. To evaluate environmental changes, the GA release medium's pH values were also tracked at predetermined intervals.
 Three different kinds of scaffolds were submerged in 20 milliliters of PBS with 0.4 milligrams of enzyme per milliliter for tests of degradation. Following a 60-day incubation period at 37 °C, the scaffolds were freeze-dried and weighed. Scanning electron microscopy was used to assess structural deterioration by observing morphological alterations.

**1.5 Cell Culture and Cytocompatibility**

The scaffolds' capacity for osteogenic development and cytocompatibility were evaluated using bone marrow mesenchymal stem cells (BMSCs). Trypsin detachment was used to transit BMSCs after they were extracted from rats and cultivated to 100% confluency. Subsequent tests were conducted using cells from the second passage.
 BMSCs were seeded onto various scaffolds in 48-well plates at a density of 2 × 10⁴ cells per well, and they were incubated for 1, 3, and 7 days. Living and dead cells were fluorescently stained using a Calcein AM/propidium iodide (PI) live-dead staining kit (Solarbio, Beijing, China). An Olympus fluorescent microscope was then used to analyse the cell morphology. Cell viability was assessed using the CCK-8 assay. Specifically, BMSCs were cultivated at a density of 4 × 10⁴ cells per well on a variety of scaffolds and incubated for 1, 3, and 7 days under standard conditions. Following incubation, a CCK-8 solution was added to the culture medium. A microplate reader (Bio-Rad 680) was used to detect the absorbance at 450 nm after an additional hour of incubation at 37 °C.

**1.6 Antioxidative Activity**

Methanol was used to dissolve 1,1-diphenyl-2-picrylhydrazyl (DPPH), resulting in a 1 mmol/L solution. Each scaffold received four millilitres of the DPPH solution before being placed in a dark area to react. The absorbance at 517 nm was measured using a multifunctional microplate reader (Tecan Infinite M200, Switzerland) after aliquots of the solution were obtained at predefined intervals (0, 20, 40, 60, 80, 100, and 120 minutes).

**1.7 Antioxidant Assay**

BMSCs were seeded into 24-well Transwell inserts at a density of 4 × 10⁴ cells per well. The cells in the bottom compartment were treated with 100 μM hydrogen peroxide (H_2_O₂) after a 12-hour incubation period. Multiple scaffolds were simultaneously set up in the upper rooms. Inserts with their top chambers empty made comprised the control group. After a 24-hour period, the amounts of reactive oxygen species (ROS) in the BMSCs were measured using a ROS assay kit (Yeasen, China). The vitality of BMSCs was further investigated using live/dead staining and an inverted fluorescence microscope. Additionally, the expression of genes associated with antioxidants was evaluated in BMSCs.
 24-well plates were seeded with RAW264.7 macrophages, which were acquired from the Chinese Academy of Sciences' Shanghai Cell Bank, on glass coverslips. After the macrophages achieved 60–70% confluency, the cells were co-cultured for 24 hours with various scaffolds. A ROS assay kit was then used to assess the ROS levels in the macrophages.

**1.8 Identification of the Macrophage Phenotype**

To produce an inflammatory environment, interferon-gamma (IFN-γ, 20 ng/mL) and lipopolysaccharide (LPS, 100 ng/mL) were added to the culture medium. Healthy RAW264.7 macrophages were co-cultured with different scaffolds for a whole day. Following incubation, the cells were fixed with 4% paraformaldehyde and permeabilized with Triton X-100 (Beyotime Biotechnology, Shanghai, China). The polarised macrophages were treated with a blocking solution for 30 minutes to decrease nonspecific binding, and then they were incubated with primary antibodies for the whole night. The cells were then exposed to fluorescent secondary antibodies for two hours at room temperature. The two primary macrophage phenotypes, iNOS (M1 marker) and CD206 (M2 marker), were seen using fluorescence microscopy.

Using flow cytometry, the ratios of M1 and M2 macrophages were determined. To prevent nonspecific antigen binding, cells were collected into Eppendorf (EP) tubes after four days of scaffold co-cultivation, centrifuged, and blocked for 30 minutes using 1% bovine serum albumin (BSA). The cells were then stained with phycoerythrin (PE)-conjugated CD206 and allophycocyanin (APC)-conjugated CCR7 for an hour in the dark. Isotype controls included rat IgG2a,κ conjugated with PE, rat IgG2a,κ conjugated with APC, and rat IgG2a,κ conjugated with fluorescein isothiocyanate (FITC). After staining, 100 μL of the cell suspension was transferred to a 96-well plate and evaluated using a Guava flow cytometer (Millipore, USA). To process the data, RAW264.7 and Guava software 3.1.1 were used.Seven cells grown on a 12-well plate made comprised the control group.

Additionally, real-time polymerase chain reaction (RT-PCR) was used to evaluate the expression levels of the M1 macrophage marker CCR7 and the M2 marker CD206. Primer sequences are included in **Table S2**.
 The macrophage culture medium was removed and centrifuged to evaluate cytokine secretion after four days of scaffold incubation. TNF-α, IL-4, IL-6, and IL-10 levels in the supernatants were measured using ELISA kits (Anogen, Canada) in accordance with the manufacturer's instructions.

**1.9 In Vitro Angiogenic Evaluation**

Human umbilical vein endothelial cells (HUVECs) were cultivated for one, two, and three days after being seeded onto different scaffolds in 48-well plates at a density of 1×10⁴ cells per well. The cells were maintained at 37 °C in DMEM/F12 medium supplemented with 10% foetal bovine serum (FBS). The cells' vitality was assessed using a Calcein AM/propidium iodide (PI) live-dead staining kit (Solarbio, Beijing, China). Fluorescent images were captured using an Olympus fluorescence microscope.

**1.10 Wound Healing Assay**

HUVECs were seeded onto 24-well plates at a density of 2×10⁵ cells per well. Once the cells reached 90% confluence, the monolayer was scraped off using a sterile 200 μL pipette tip. The cells were either treated with PBS or extracts of several MOFs after two PBS washes. After that, the cells were grown in DMEM/F12 medium with 1% FBS added. Wound closure was examined under an Olympus optical microscope at 0 and 12 hours, and the closure ratio was computed using ImageJ software.

**1.11 Tube Formation Assay**

HUVECs were planted in 6-well plates and cultured with extracts from various scaffolds for a full day in order to assess the scaffolds' angiogenic potential. After treatment, 96-well plates covered with Matrigel (Corning, NY, USA) were plated with two × 10⁴ cells per well, and the cells were grown for six hours. An inverted microscope (Olympus) was used to observe and visualise the creation of tubular formations. The tube networks were analysed using ImageJ software.

**1.12 Migration Assay**

Cell mobility was measured using a 24-well Transwell system with 8 μm pore inserts. HUVECs (2×10⁴ per well) suspended in 100 μL of serum-free DMEM/F12 were used to seed the top chamber. The bottom chamber was filled with 800 microlitres of diluted scaffold extracts. The migrated cells in the bottom chamber were fixed with 4% paraformaldehyde after a 24-hour incubation period, and the non-migrated cells in the top chamber were extracted using cotton swabs. An optical microscope (Olympus, Japan) was used to acquire photographs of the cells after they had been stained with 0.1% crystal violet for 30 minutes.

**1.13 Vascularization Genes and Protein Expression Assay**

For 48 hours, HUVECs and various scaffolds were co-cultured by indirect contact. Real-time quantitative polymerase chain reaction (RT-qPCR) was used to measure the expression levels of genes linked to vascularization, such as cluster of differentiation 31 (CD31) and vascular endothelial growth factor (VEGF). Standard procedures were followed for performing the Western blot analysis. **Tables S2** and **S3** provide a list of the primer sequences and primary antibodies utilized in this experiment.
 After being treated with various MOFs for 48 hours, HUVECs were fixed in 4% paraformaldehyde (PFA) for 15 minutes and then washed three times with PBS. The cells were then blocked in 1% bovine serum albumin (BSA) for 30 minutes at room temperature to reduce nonspecific binding. After that, the cells were incubated at 4 °C for the whole night with primary antibodies that target VEGF and CD31. After being rinsed with PBS, the cells were exposed to fluorescently tagged secondary antibodies for an hour at room temperature. Finally, the cell nuclei were stained with DAPI for five minutes.A fluorescence microscope (Olympus, Japan) was used to obtain fluorescent images, which were then analysed using ImageJ software to determine the levels of protein expression.

**1.14 In Vitro Neurogenic Evaluation**

Schwann cell-96 (RSC-96) were seeded with various scaffolds in 48-well plates at a density of 2 × 10⁴ cells per well, and they were then incubated for 48 hours. Living and dead cells were fluorescently stained using a Calcein AM/propidium iodide (PI) live-dead staining kit (Solarbio, Beijing, China). Cell viability was assessed using the CCK-8 assay. The cells were frozen in 4% PFA for 30 minutes, permeabilized with 0.1% Triton X-100 for 5 minutes, and then treated with Rhodamine Phalloidin (RP, Solarbio, China) for 2 hours in the dark to observe the cellular morphology of RSC-96 cells grown on different scaffolds. An Olympus fluorescence microscope was used to take pictures after 40 minutes of DAPI nuclear staining.

**1.15 Migration Assay**

RSC-96 cells were seeded onto 24-well plates at a density of 2×10⁵ cells per well. Once the cells reached 90% confluence, the monolayer was scraped off using a sterile 200 μL pipette tip. After two PBS washes, the top chamber was filled with various scaffolds, and the 0.4 μm pore inserts (Labselect, China) were inserted. Wound closure was examined under an Olympus optical microscope at 0 and 12 hours, and the closure ratio was computed using ImageJ software.

**1.16 Immunofluorescence Staining**

Following a 12-hour treatment with various scaffolds, the RSC-96 cells underwent two PBS washes before being fixed for 15 minutes in 4% paraformaldehyde (PFA) and blocked for 30 minutes at room temperature in 1% bovine serum albumin (BSA). After that, the cells were treated for the whole night at 4 °C with anti-NF-200 and S100 antibodies. The samples were treated with secondary antibody for one hour at room temperature after a PBS washing. DAPI staining was applied to the cells for five minutes. A fluorescent microscope was used to take the pictures, and ImageJ software was used for analysis. A list of the main antibodies used in this study is provided in **Table S3**.

**1.17 RT‒qPCR Analysis and Enzyme-linked Immunosorbent Assay In Vitro**

RSC-96 cells and different scaffolds were co-cultured via indirect contact for five days. Genes associated with vascularization, including MBP, PMP22, and P0, were measured for expression levels using real-time quantitative polymerase chain reaction (RT-qPCR). The primer sequences and primary antibodies used in this experiment are included in **Table S2**.
 The supernatants were collected to quantify the release of cytokines during a 4-day incubation period with the scaffolds. NGF and BDNF levels in the supernatants were measured using ELISA kits (Anogen, Canada) in accordance with the manufacturer's instructions.

**1.18 Osteogenic Differentiation In Vitro**

Bone marrow mesenchymal stem cells (BMSCs) were tested for osteogenic differentiation using alizarin red S (ARS) and alkaline phosphatase (ALP) staining. ALP staining was performed on days 7 and 14 of incubation, followed by ARS staining on days 14 and 21. Calcium nodules and stained (positive) cells were visible under an optical microscope. To measure ARS staining, 10% cetylpyridinium chloride was added to each well, and the absorbance at 600 nm was noted. The level of ALP activity was measured using an alkaline phosphatase test kit (Beyotime Biotechnology, Shanghai, China).

RT-qPCR was used to assess the relative expression of important osteogenic genes in treated BMSCs, such as runt-related transcription factor 2 (RUNX2), collagen type I (COL-1), osteocalcin (OCN), and alkaline phosphatase (ALP). **Table S2** contains primer sequences. Each sample included 50 μg of protein, which was loaded onto an SDS-PAGE gel, electrophoresed, and then transferred to a nitrocellulose membrane for protein expression analysis. After blocking the membranes for an hour at room temperature, primary antibodies were incubated with them for the whole night at 4 °C. For two hours at room temperature, the proper secondary antibodies (1:20,000; ABclonal, Wuhan, China) were administered. An ECL reagent (Yeason, Shanghai, China) was used to observe protein bands, and ImageJ software was used to conduct densitometric analysis of non-saturated bands. **Table S3** contains a list of the primary antibodies utilized in this investigation.

**1.19 Indirect Co-culture Assessment In Vitro**

To assess how different scaffolds affect the interactions between distinct functional cell types, indirect co-culture models were developed. RAW264.7, HUVECs, and RSC-96 cells were individually seeded on distinct scaffolds (n=3) and cultured for a predetermined period. Subsequently, the culture supernatants were harvested, centrifuged, and stored for further analysis. Next, HUVECs, RSC-96 cells, or BMSCs were seeded at a density of 4 × 10⁴ cells per well in 12-well plates (n = 3) and cultured for 72 hours in a 1:1 (v/v) mixture of the medium collected from the cell-scaffold cultures and fresh, corresponding medium. Finally, the cells were collected and subjected to downstream analyses, including RT-qPCR, immunofluorescence staining, cytoskeletal staining, and transwell migration assays, as described previously.

**1.20 RNA Sequencing (RNA-seq) and Bioinformatic Analyses**

Total RNA was isolated from RSC-96 cells treated with 40 μg/mL GAs or PBS (control) using TRIzol reagent. The RNA concentration was determined with the NanoDrop ND-1000 Assay Kit, and poly(A) RNA was subsequently enriched using oligo(dT) beads. An RNA library was then constructed with the KAPA Stranded RNA-Seq Library Prep Kit, and sequencing was performed using an Illumina NovaSeq 6000 platform. Raw sequencing data underwent stringent quality control prior to quantitative analysis. Differentially expressed genes and perturbed signaling pathways between the groups were identified via Gene Ontology (GO) gene set and Kyoto Encyclopedia of Genes and Genomes (KEGG) pathway enrichment analyses.

Total RNA was isolated with TRIzol reagent from BMSCs treated with PLA/GA/MgCu-MOF or PBS (control), followed by identical downstream processing and bioinformatic analyses.

**1.21 Detection of Key Pathway-associated Genes In Vitro**

Following a 72-hour treatment with GA, total protein was extracted from RSC-96 cells. In parallel, BMSCs were exposed to PLA/GA/MgCu-MOF for 10 days before total protein extraction. Western blot analysis was subsequently performed according to standard protocols. A comprehensive list of the primary antibodies used in this study is provided in **Table S3**.

**1.22 Determination of Index in Mitochondria**

The JC-1 test kit (Beyotime, China) was used to measure the mitochondrial membrane potential. Additionally, the ATP Assay Kit (Solarbio, China) was used to measure the amount of ATP.

**1.23 In Vivo Bone Regeneration Evaluation**

This animal study was approved by Shandong First Medical University's Ethics Committee, and all procedures were conducted in accordance with the moral guidelines for animal care. Thirty-six male Sprague Dawley (SD) rats, weighing about 300 g and six weeks of age, were purchased from the Experimental Animal Centre. In short, each rat was given three successive intramuscular injections of methylprednisolone (MPS; Pfizer, USA) at 24-hour intervals after receiving two intraperitoneal injections of lipopolysaccharide (LPS) at a concentration of 10 mg/kg. The rats were given a 2% sodium pentobarbital intraperitoneal injection to put them to sleep after two weeks of osteonecrosis induction. A medical drill was used to generate a cylindrical bone defect (3 mm in diameter) in the distal femur after the surgical site had been cleaned and shaved. Before implantation, the scaffold's dimensions were carefully changed to fit the size of the defect. Sutures were then used to seal the surgical wounds, and the locations were suitably noted for further identification.

**1.23.1 Vascular Perfusion**

According to a prior research, two rats from each group were chosen at random for vascular perfusion tests at two and four weeks after implantation. We gathered four femoral condylar specimens. The rats were given anesthesia, had their abdominal hair removed, and had their abdominal cavity, aorta, and inferior vena cava exposed via a midline incision. The arteries were clamped proximally after being thoroughly dissected. The inferior vena cava and proximal abdominal aorta were tied off, and a 1.5 mm infusion tube was inserted and fastened in the distal abdominal aorta. Suction was used to remove the blood and perfusion fluids.The rodents were put to death after a perfusion channel had been established. 400 U/L of heparinized saline was infused into the lower limbs until the venous outflow was evident. The hind limbs were then fixed by perfusing the arteries with 10% formaldehyde. Using an automated syringe pump, MICROFIL® MV-117 perfusion solution was made in accordance with the manufacturer's instructions and injected into the hind leg vessels at a rate of 2 mL/min. Each rat received a total volume of 50 mL. After being harvested, the femurs were decalcified in a 10% EDTA solution for two months and preserved in 4% paraformaldehyde for seventy-two hours.

**1.23.2 Micro-CT Assessment**

Rats were killed 4 and 8 weeks after implantation, and the femurs were removed and stored in 10% formalin for analysis. The vascular and bone growth within the scaffolds were examined using micro-computed tomography (Micro-CT; VivaCT80, SCANCO Medical AG, Switzerland). 385 mA, 65 kV, and 1 mm Al filters were the imaging parameters. The reconstructed images were processed using NRecon software, and the analysis was done using VG Studio Max 2.1. The quantitative features that were calculated and statistically analysed were trabecular number (Tb.N), vascular volume percentage (vascular volume/total volume, BV/TV), trabecular thickness (Tb.Th), trabecular separation (Tb.Sp), bone volume/total volume (BV/TV), and mean vessel surface.

**1.23.3 Histologic Analysis**

Rats were put to death by intraperitoneal injection of sodium pentobarbital at 1, 4, and 8 weeks after implantation. The scaffold-containing femoral flaws were removed and preserved in 4% paraformaldehyde. The samples were sectioned using a Leica RM2016 rotary microtome and imbedded in paraffin after a 45-day decalcification period. Van-Gieson (V-G) staining (Leagene, China) and hematoxylin and eosin (HE) staining were used to assess the production of collagen and new bone. The expression of proteins linked to inflammation and angiogenesis was examined using immunofluorescence labeling. A NIKON DS-U3 image system (Nikon, Japan) was used to take the pictures. Immunofluorescence staining and RT-qPCR were employed to assess the expression levels of the target proteins and their corresponding genes. **Tables S2** and **S3** provide a list of the primer sequences and primary antibodies utilized in this experiment.

**1.24 Statistical Analysis**

The mean±standard deviation (SD) was used to display all data. The SPSS 26.0 software's  t-test and one-way analysis of variance (ANOVA) were used for the statistical studies. P-values less than 0.05 indicated that a difference was statistically significant.

**2.Supplementary figures and tables**


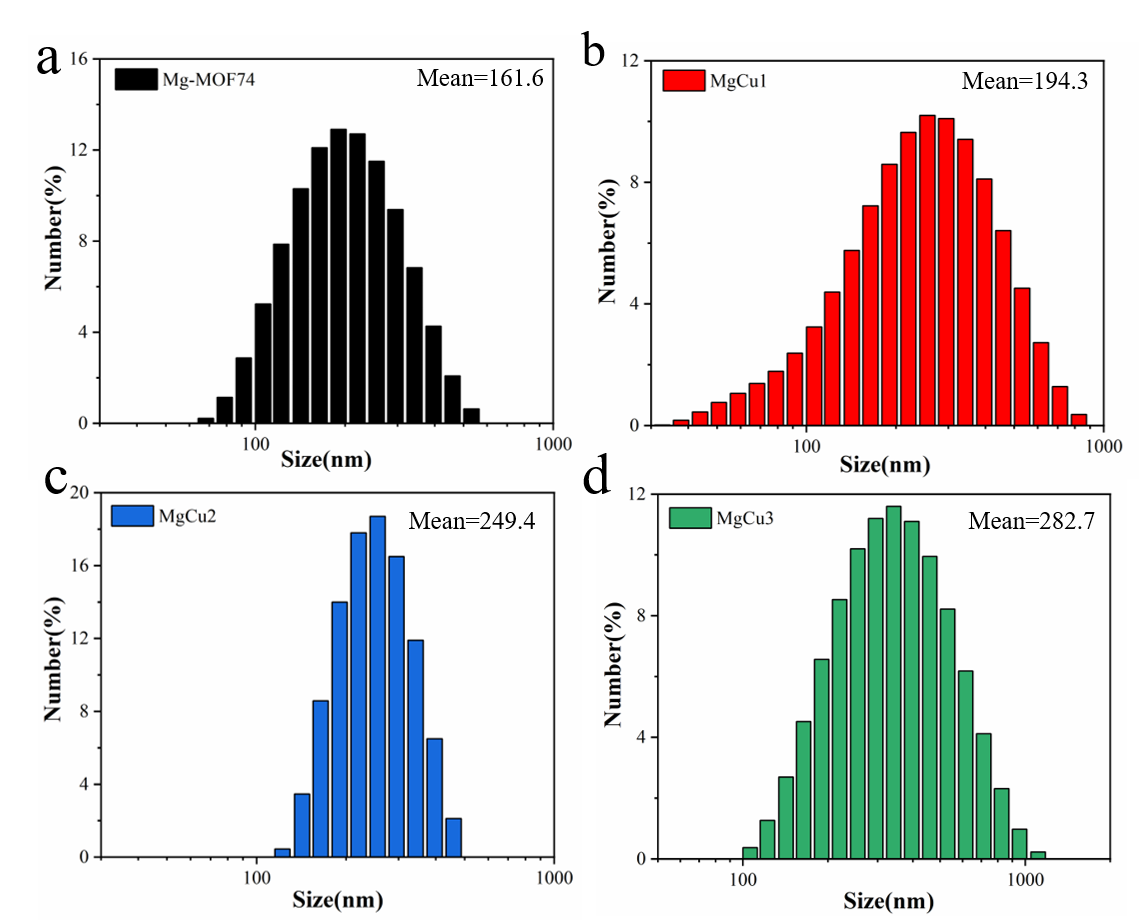


**Figure S1. (a-d)** **Hydrodynamic size distribution of different samples in PBS.**





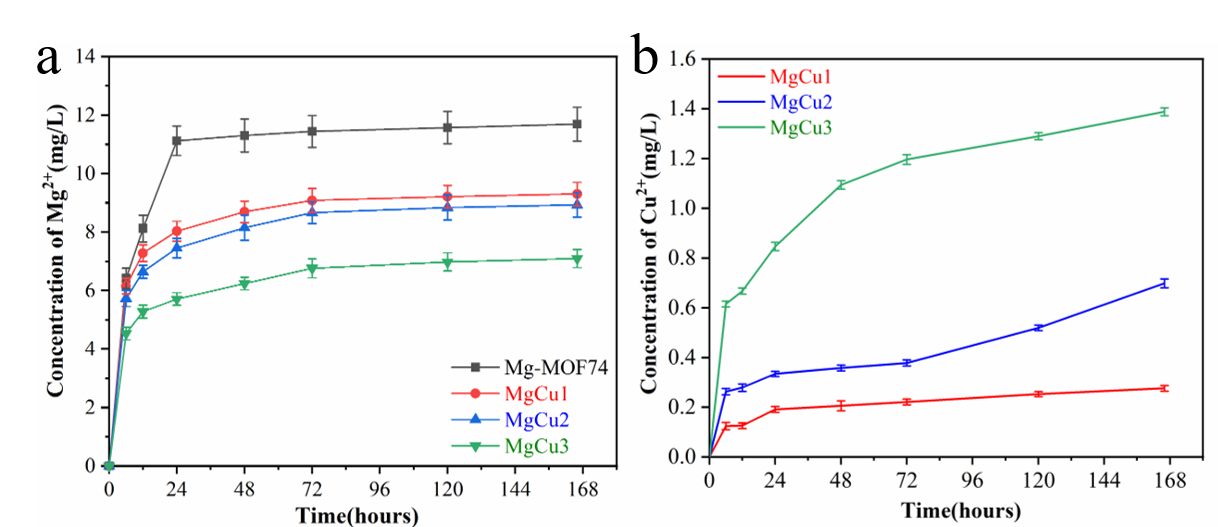
**Figure S2. EDS results of different samples.**


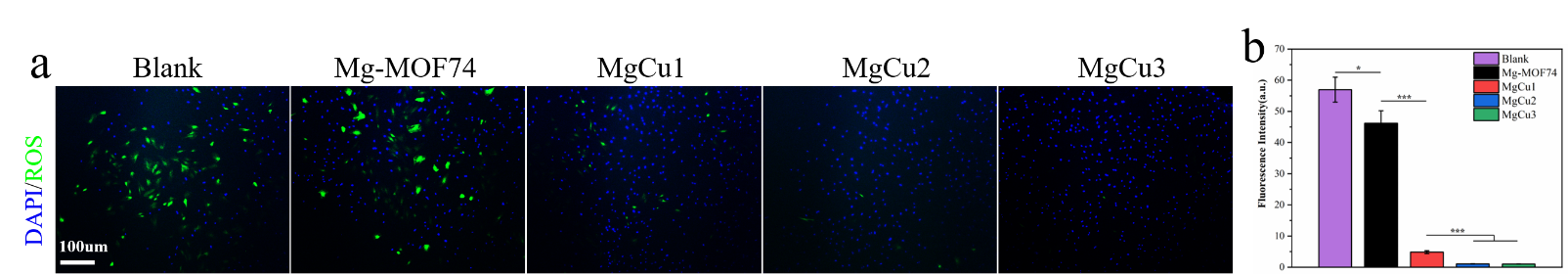
**Figure S3. The released (a) Mg^2+^ and (b) Cu^2+^ in physiological (pH 7.4) microenvironment.**

**Figure S4. (a)Fluorescence images of Hoechst 33342 (blue) and DCFH-DA (green) in BMSCs after 24 hours with 100 μM H₂O₂ under various treatments. (b)ROS fluorescence intensity in BMSCs. (n = 3; **P* < 0.05, ***P* < 0.01, ****P* < 0.001).**


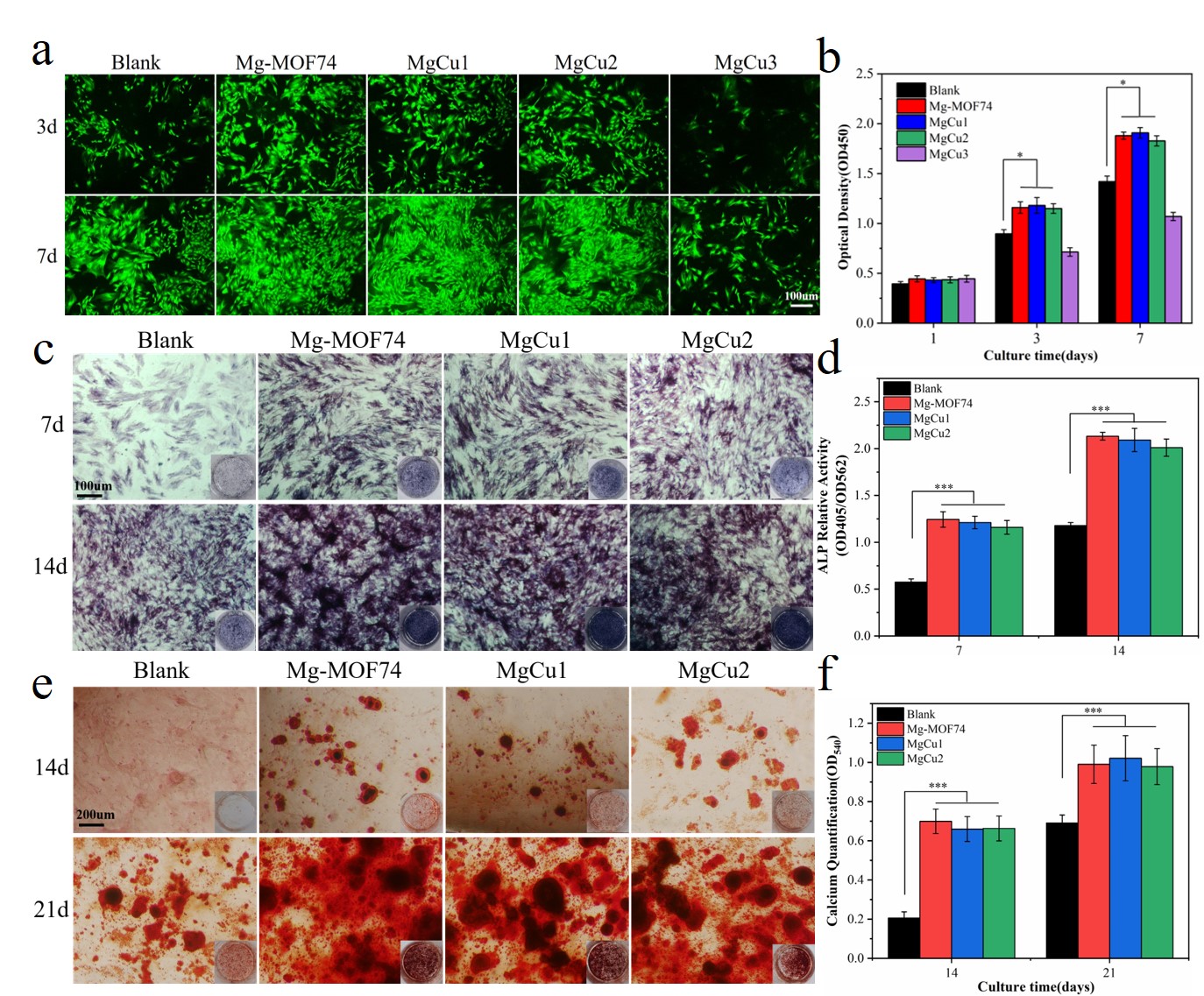


**Figure S5.** **(a)Live/dead staining for BMSCs after treatment** **with different** **MOFs. (b)** **Absorbance (450 nm) of BMSCs cultured with various MOFs for 1, 3 and 7 days. (c) Alkaline phosphatase (ALP) staining. (d) Quantitative analysis of ALP staining results. (e) Alizarin red staining. (f) Quantitative analysis of Alizarin red staining results. (n = 3; **P* < 0.05, ***P* < 0.01, ****P* < 0.001).**


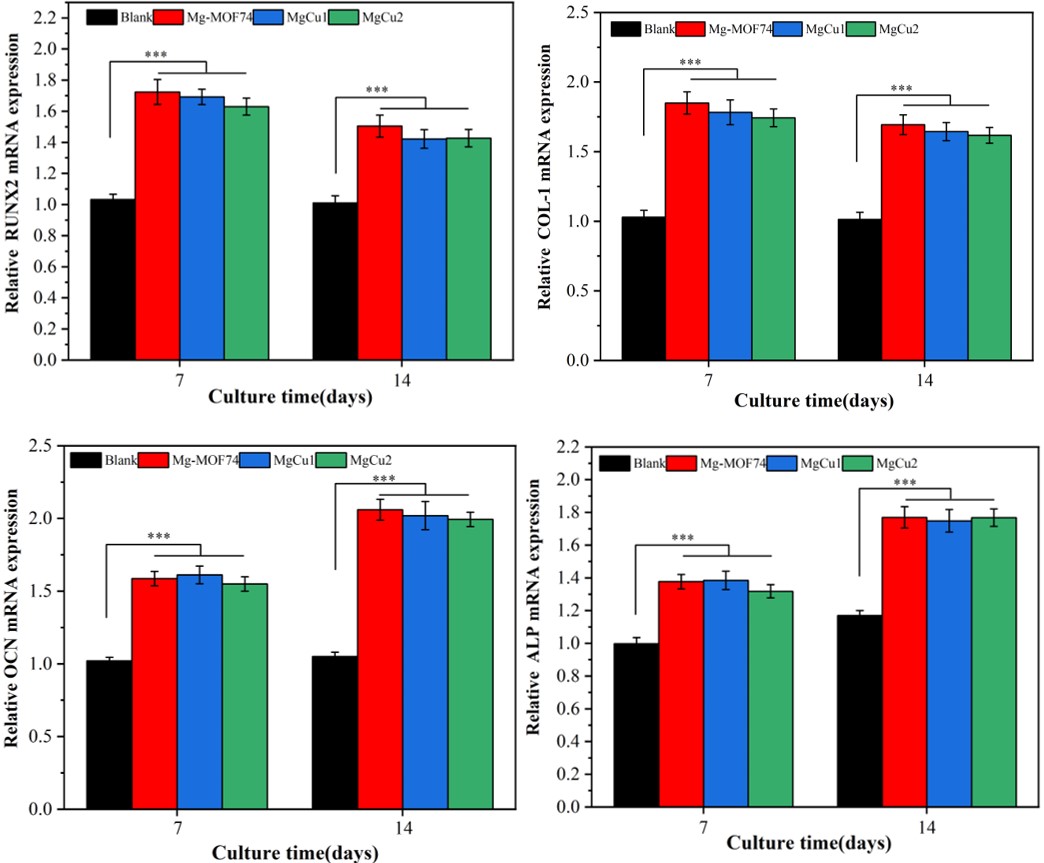


**Figure S6. RT‒qPCR analysis of the expression levels of osteogenic-related markers, including Runx2 (a), Col-I (b), Ocn(c) and ALP(d), in BMSCs cultured with different MOFs on days 7 and 14. (n = 3; **P* < 0.05, ***P* < 0.01, ****P* < 0.001).**


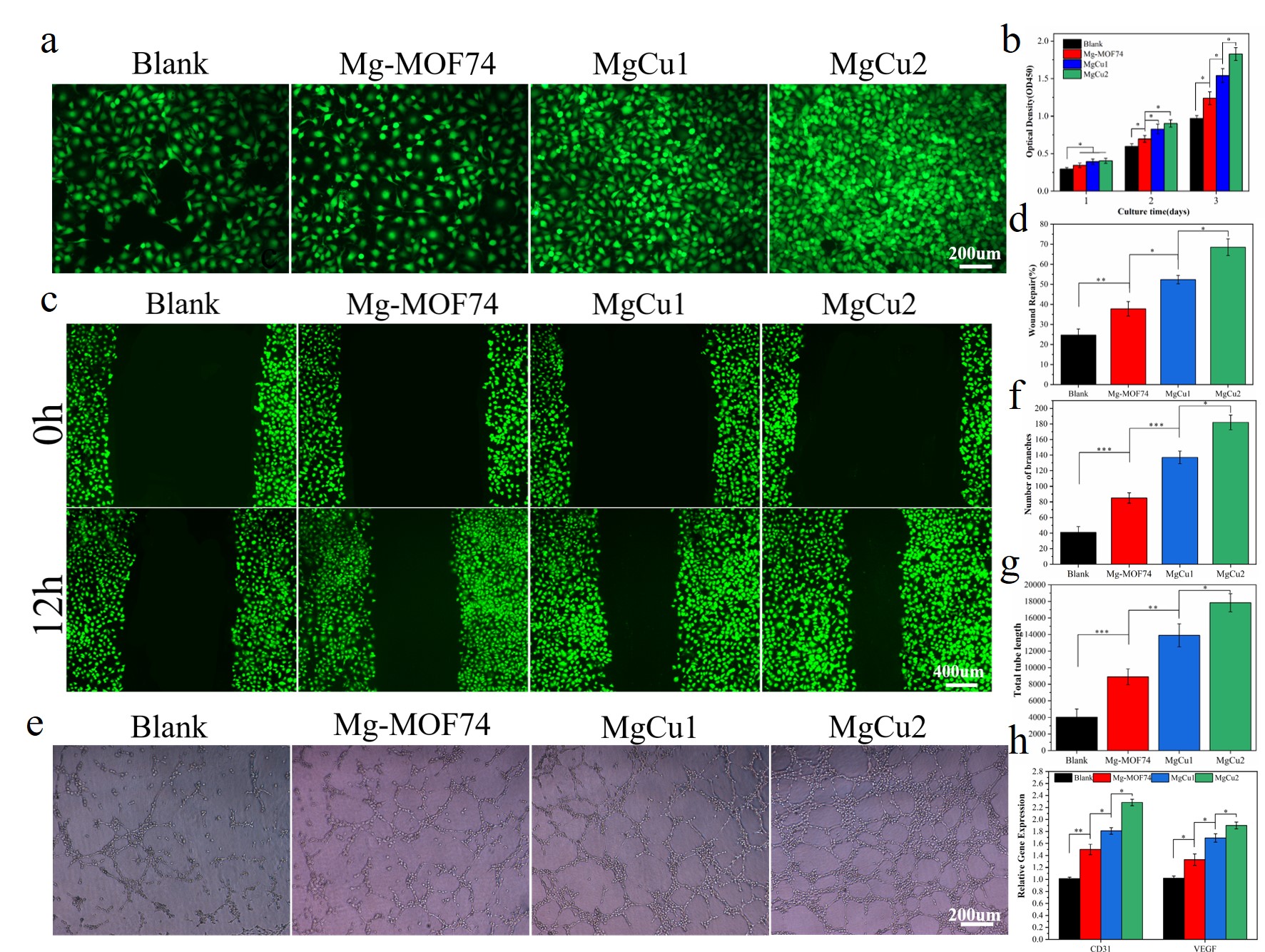
**Figure S7.** **(a)** **Live/dead staining for** **HUVECs after treatment with different MOFs for 2 days. (b) Absorbance (450 nm) of HUVECs cultured with various MOFs for 1, 2 and 3 days. (c,d)** **Representative images of wound healing and wound closure ratio in HUEVCs treated with different MOFs at 0h and 12h.** **(e)Tube formation assay of HUVECs treated with different MOFs.** **(f,g)Quantitative analysis of the number of branches and total tube length. (h) RT‒qPCR analysis of CD31 and VEGF expression in HUVECs treated with different MOF. (n = 3; **P* < 0.05, ***P* < 0.01, ****P* < 0.001).**

**
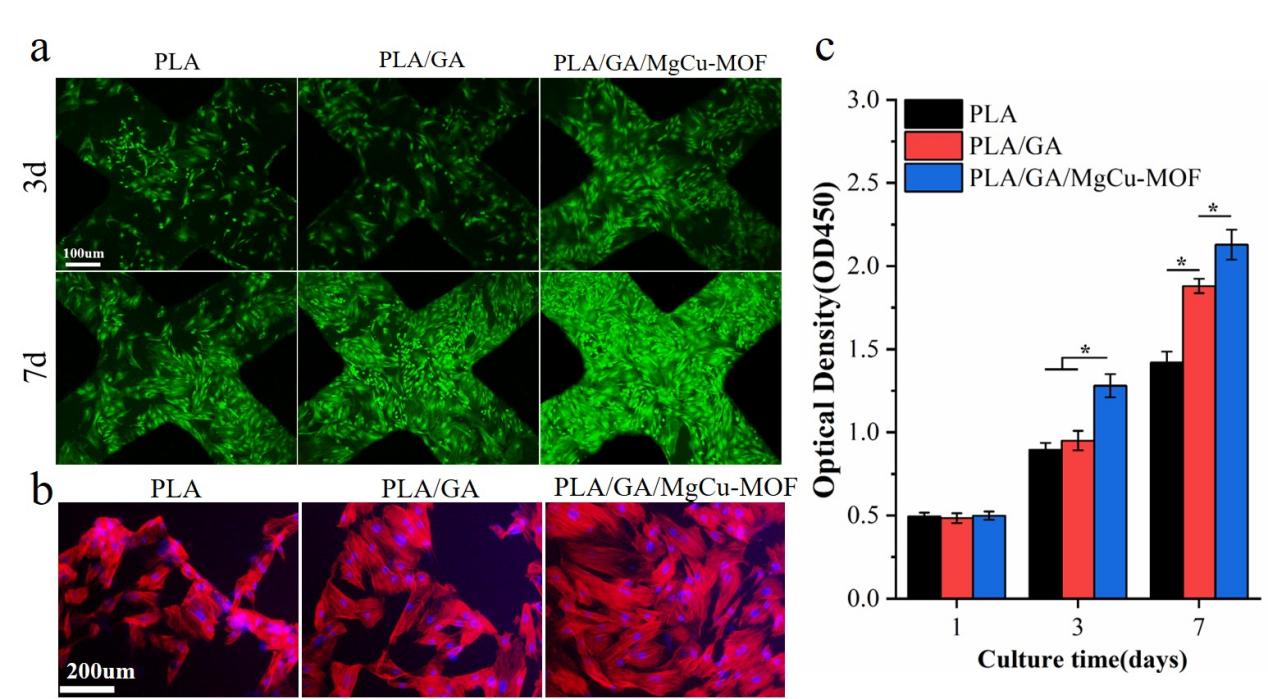
**

**Figure S8.** **(a)Live(green)/dead(red) staining for BMSCs after treatment with different scaffolds. (b)High resolution laser scanning confocal microscopy images of cytoskeleton (red, phalloidin) and nuclei (blue, DAPI), showing the BMSCs cultured on different scaffolds for 4 days. (c) Absorbance (450 nm) of BMSCs cultured with various scaffolds for 1, 3 and 7 days. (n = 3; **P* < 0.05, ***P* < 0.01, ****P* < 0.001).**


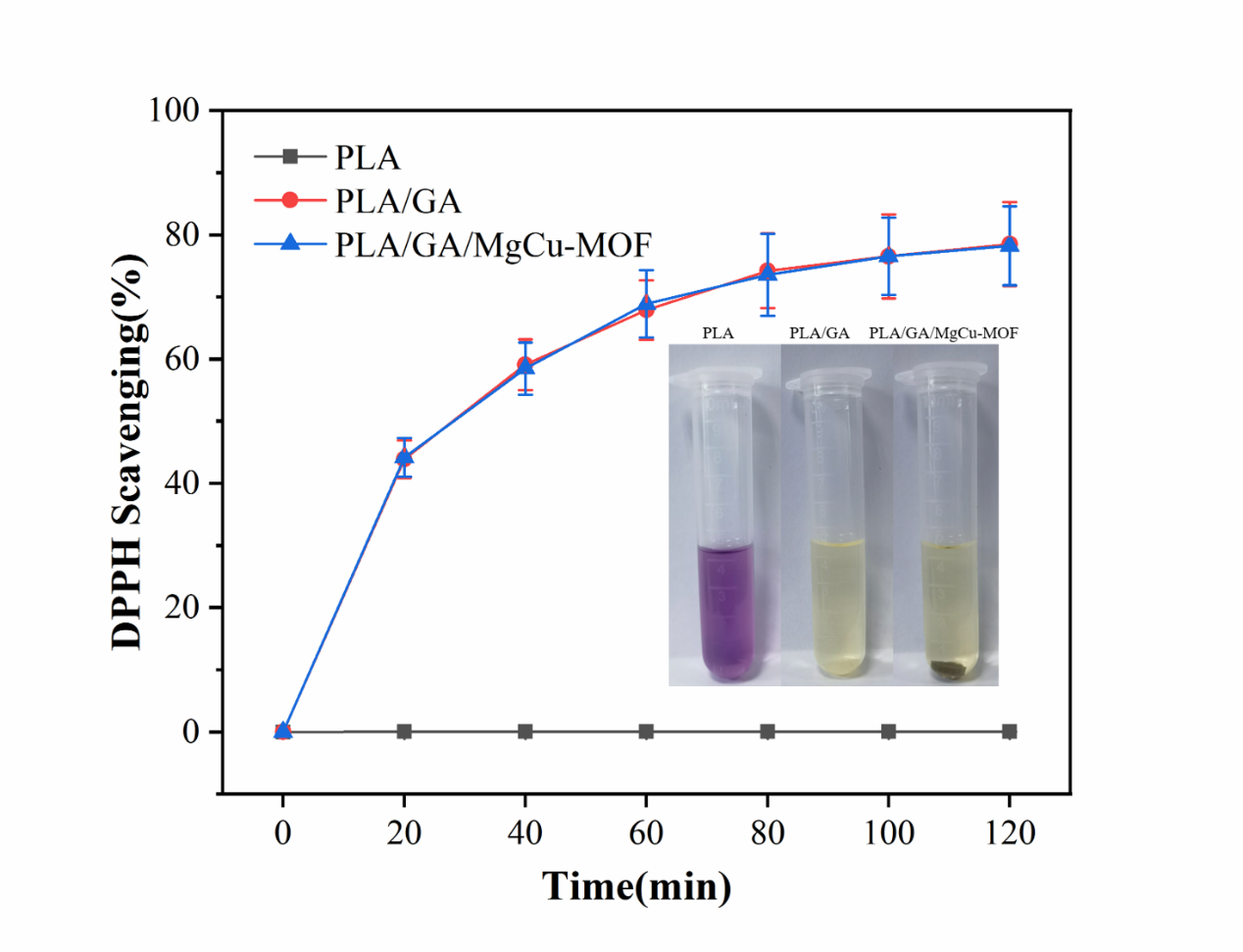
**Figure S9.** **The degradation percentage of DPPH.**

**
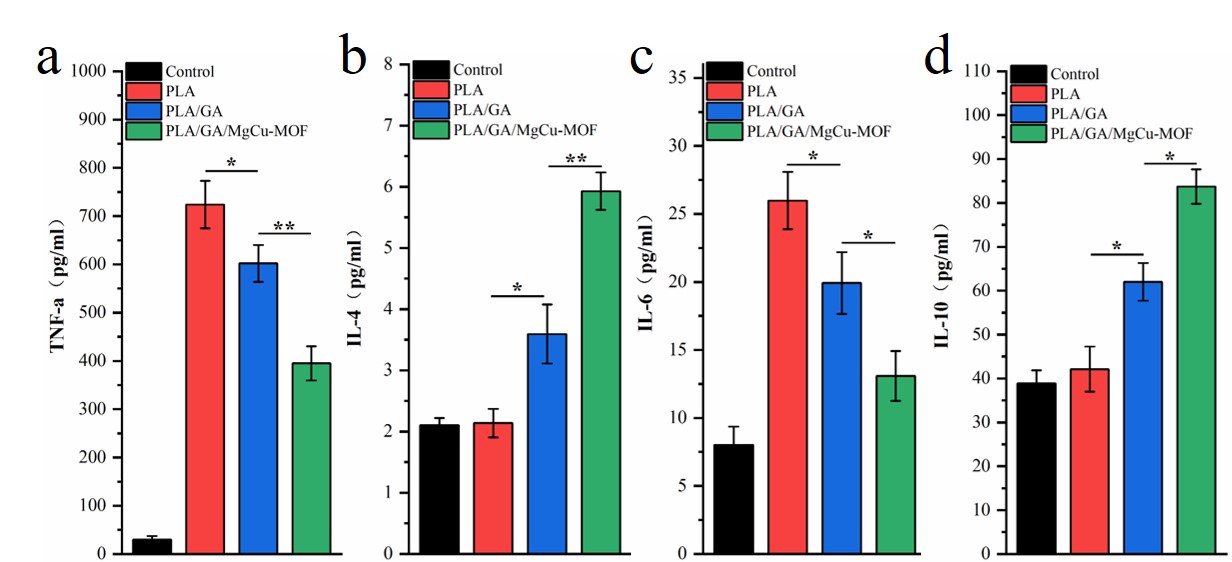
**

**Figure S10. (a–d) Elisa results of TNF-ɑ, IL-4, IL-6, and IL-10 respectively.** **(n = 3; **P* < 0.05, ***P* < 0.01, ****P* < 0.001).**


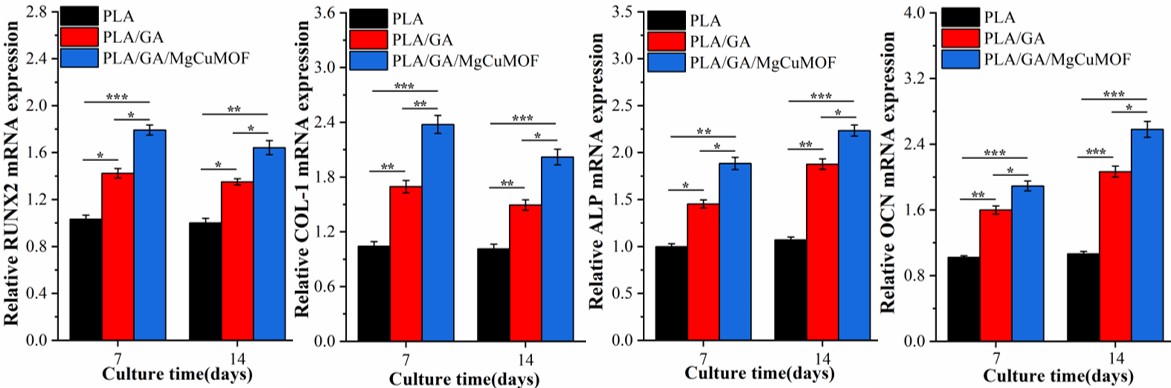


**Figure S11.** **RT-qPCR analysis of** **Runx2, Col-I, OCN, and ALP gene expression in** **BMSCs with different treatments for 7 and 14 days. (n = 3; **P* < 0.05, ***P* < 0.01, ****P* < 0.001).**

**Figure S12.** **Gene Ontology (GO) analysis of DEGs**
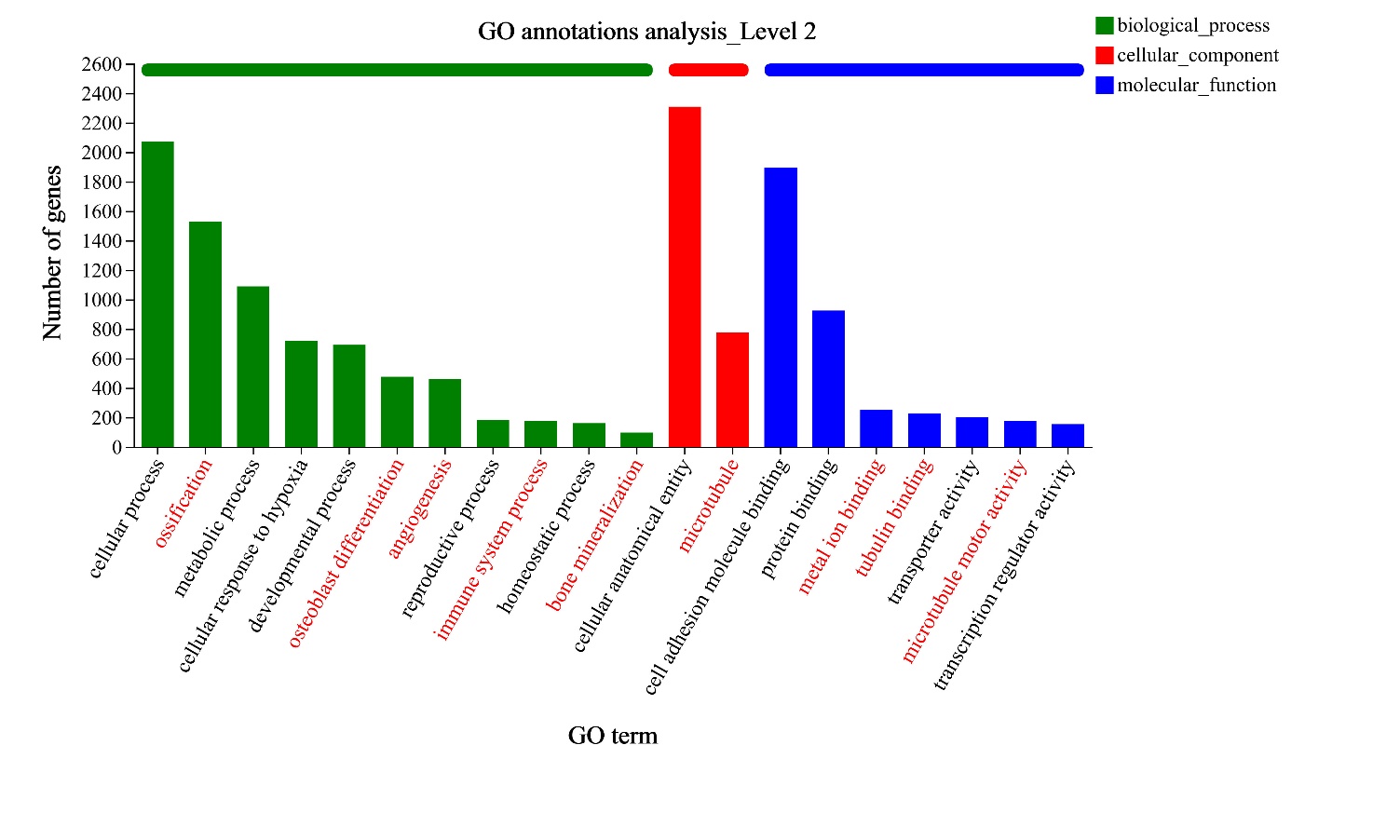
 **between GA and Control groups.**

**
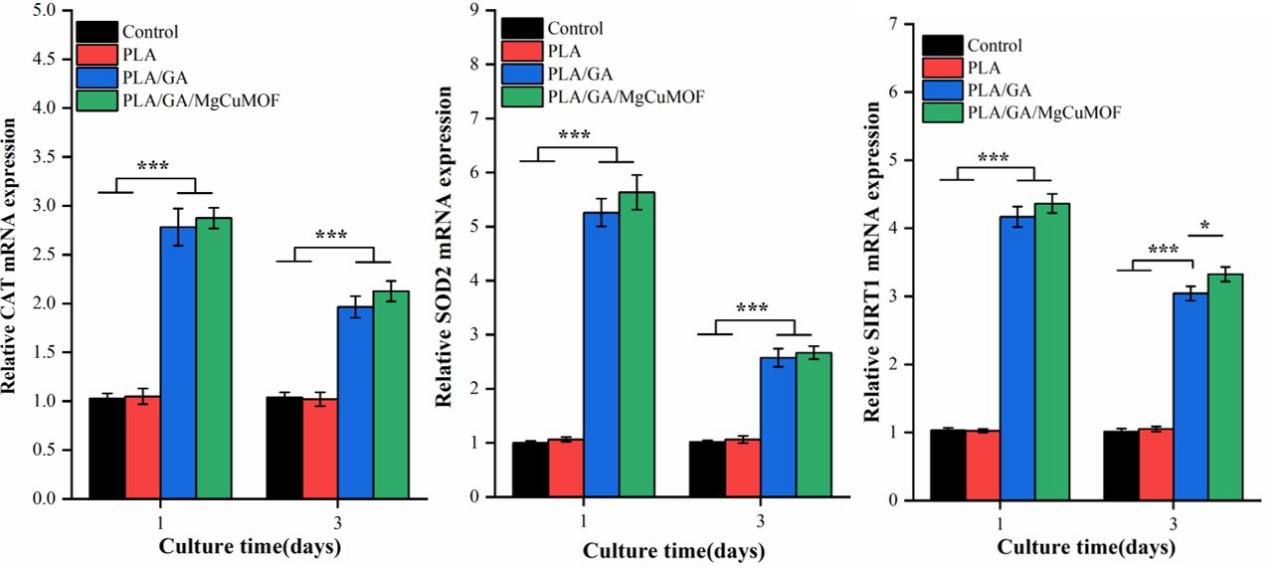
**

**Figure S13.** **Expression of antioxidant genes(CAT,SOD2 and SIRT1) in BMSCs treated with H_2_O_2_ and cultured on different scaffolds. (n = 3; **P* < 0.05, ***P* < 0.01, ****P* < 0.001).**


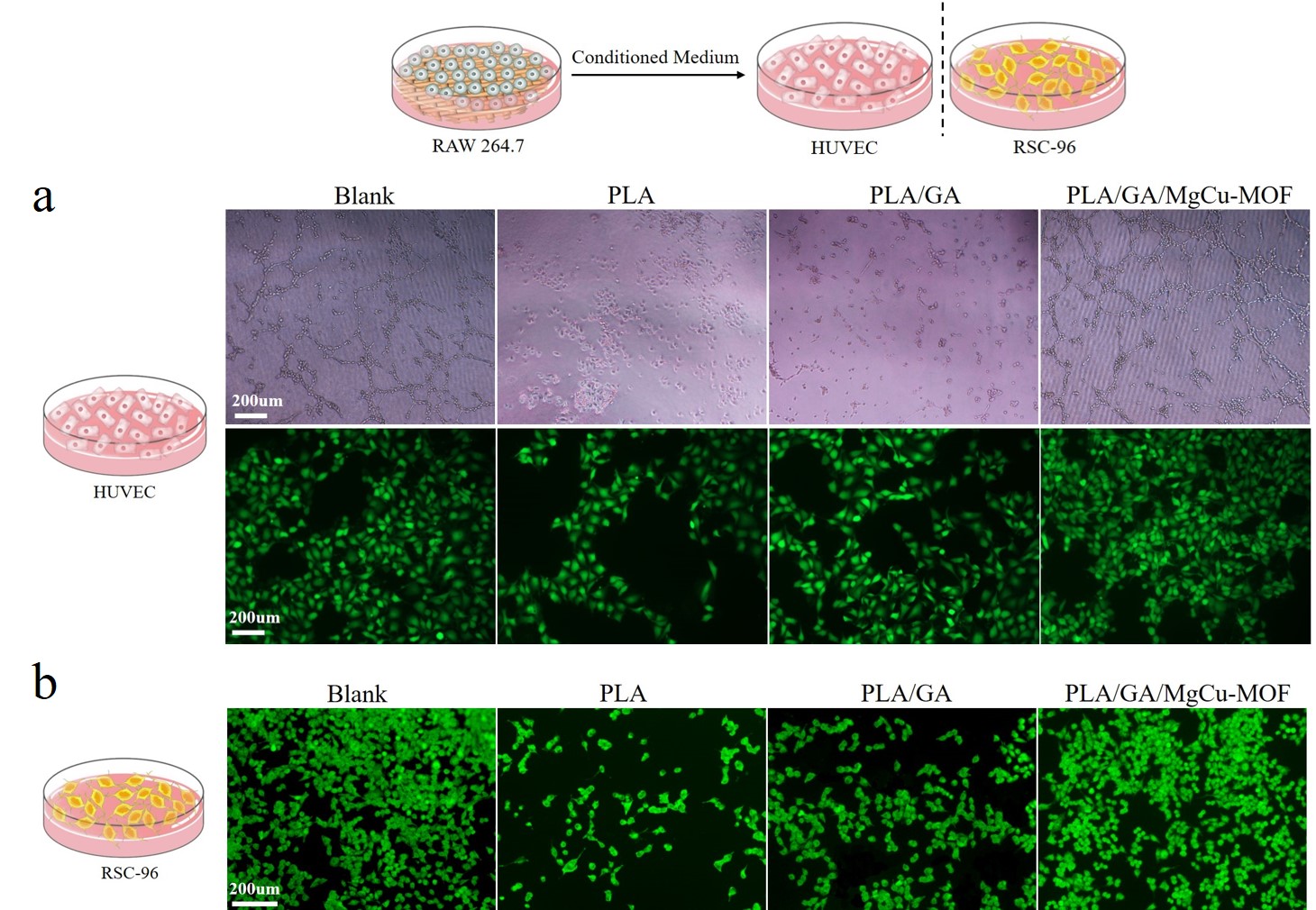


**Figure S14. (a) Tube formation assay and live/dead staining of HUVECs, (b) Live/dead staining of RSC-96 in the conditioned medium from RAW 264.7 treated with various scaffolds.**


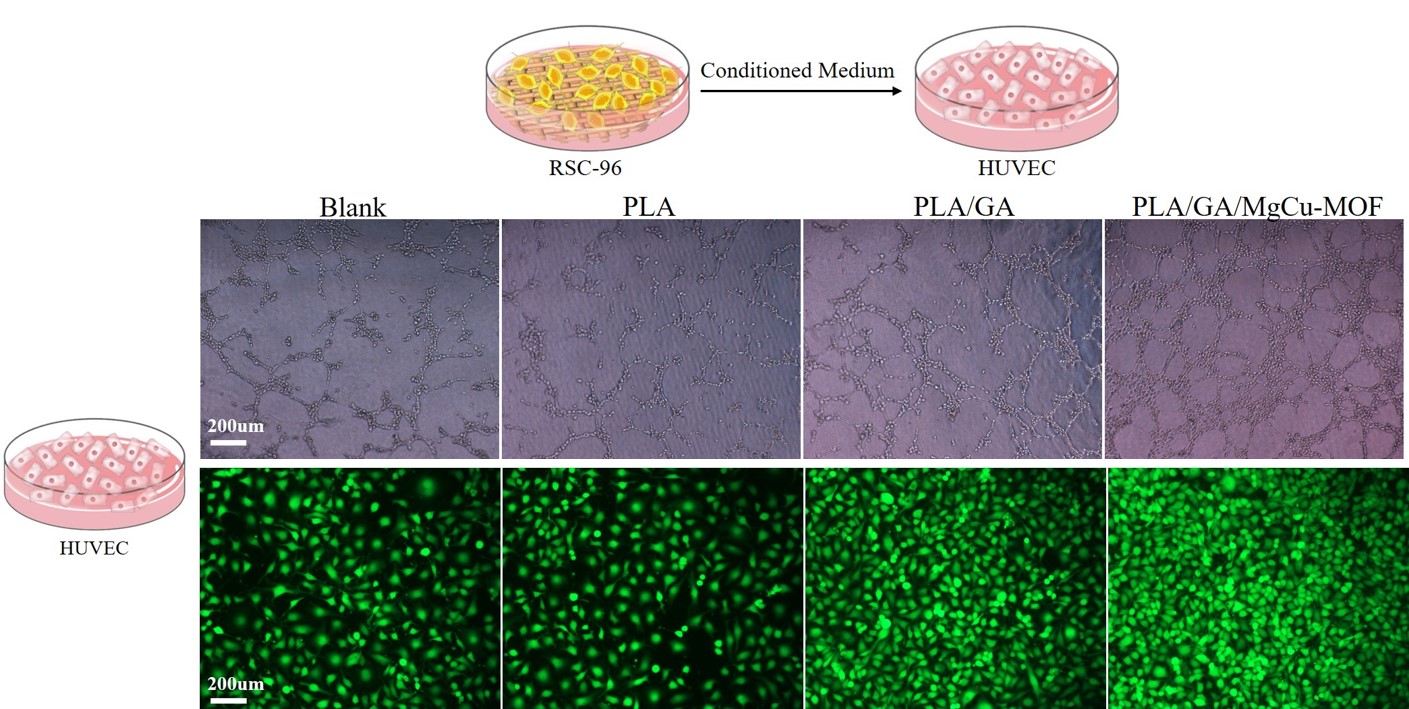

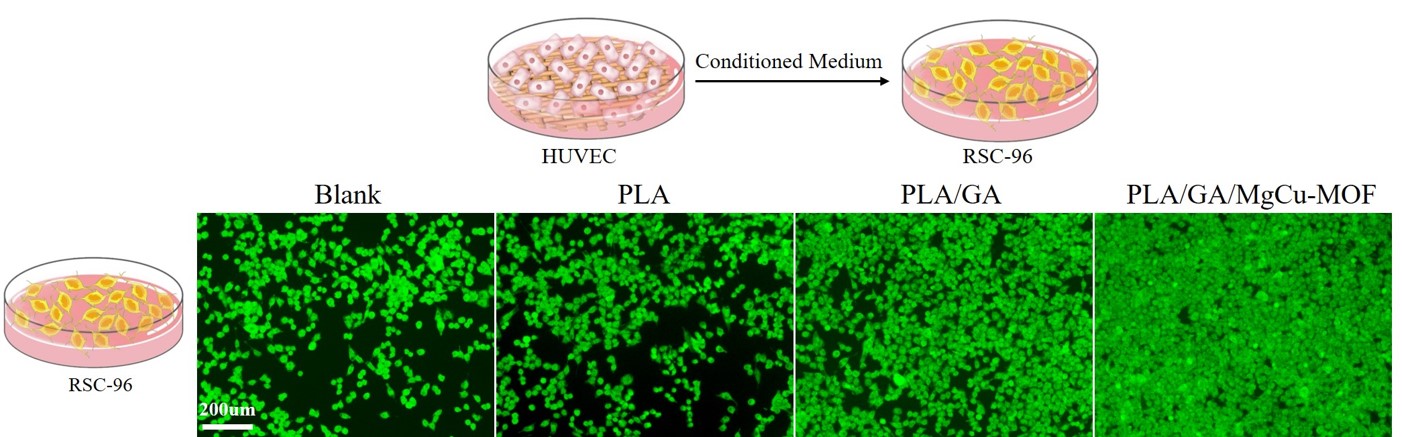
 **Figure S15.** **Live/dead staining of RSC-96 in the conditioned medium from HUVECs treated with various scaffolds.**

**Figure S16.** **Tube formation assay and** **live/dead staining of HUVECs in the conditioned medium from RSC-96 treated with various scaffolds.**

| Samples | DHTA/mg | PVP/mg | Mg(NO_3_)_2_·6H_2_O/mg | Cu(NO_3_)_2_·3H_2_O/mg |
| --- | --- | --- | --- | --- |
| Mg-MOF74 | 79.2 | 50 | 333.33 | 0 |
| MgCu1 | 79.2 | 50 | 326.92 | 6.04 |
| MgCu2 | 79.2 | 50 | 307.69 | 24.16 |
| MgCu3 | 79.2 | 50 | 269.23 | 60.4 |

**Table S1 The chemical components of reaction solution in different samples**

**Table S2 Real-time PCR primer sequences**

| **Gene** | **Primers (F=forward, R=reverse)** |
| --- | --- |
| SIRT1-Rat | F: TGGCAAGATACTGGCATGTG |
|  | R: GGACAGAGTCCATTGTTCTGC |
| SOD2-Rat | F: CCAGGAATGGAAA AGGAGTG |
|  | R: GTAACAGCCAAACGTGACTT |
| CAT-Rat | F: AGGACATCGTGGAGAAAGCA |
|  | R: CTCTGTGCCATCCTGTTGTC |
| Runx2-Rat | F: CCGAGACCAACCGAGTCATTTA |
|  | R: AAGAGGCTGTTTGACGCCAT |
| Ocn-Rat | F: TTATTGTTTGAGGGGCCTGGG |
|  | R: ACACAACTGCAGGTCGAGTTT |
| ALP-Rat | F: CAACGTGGCCAAGAACATCA |
|  | R: CCTGAGCGTTGGTGTTGTAC |
| Col-I-Rat | F: CCCAGCGGTGGTTATGACTT |
|  | R: TCGATCCAGTACTCTCCGCT |
| β-actin-Rat | F: CTTGTGCAGTGCCAGCCTC |
|  | R: GATGGTGATGGGTTTCCCGT |
| VEGF-Human | F: CTTCAAGCCATCCTGTGTGC |
|  | R: TTTGATCCGCATAATCTGCATGG |
| CD31-Human | F: TGAGGAAAGCCAAGGCCAAG |
|  | R: GGACAGCTTTCCGGACTTCA |
| β-actin-Human | F: TGTGTCCGTCGTGGATCTGA |
|  | R: TTGCTGTTGAAGTCGCAGGAG |
| CCR7-Mouse | F: GGTGGCTCTCCTTGTCATTTTC |
|  | R: AGGTTGAGCAGGTAGGTATCCG |
| CD206-Mouse | F: TACTTGGACGGATAGATGGAGG |
|  | R: CATAGAAAGGAATCCACGCAGT |
| β-actin-Mouse | F: GTGACGTTGACATCCGTAAAGA |
|  | R: GTAACAGTCCGCCTAGAAGCA |
| MBP-Rat | F: GAGATTCACCGAGGAGAGG |
|  | R: GTGTGCTTGGAGTCTGTCA |
| PMP22-Rat | F: TGTACCACATCCGCCTTGG |
|  | R: GAGCTGGCAGAAGAACAGGAAC |
| P0-Rat | F: TGCTGCTGATTCTAGGGATGTC |
|  | R: TTCACACACGGTCTGGTTGG |
| S100-Rat | F: GTTGCCCTCATTGATGTCT |
|  | R: CTGCTCTTTGATTTCCTCC |
| NF-200-Rat | F: GTTCCGAGTGAGGTTGGACC |
|  | R: CCGCCGGTACTCAGTTATCTC |
| NGF- Rat | F: CGCTCTCCTTCACAGAGTTTT |
|  | R: GACATTACGCTATGCACCTCAG |
| TUBB3-Rat | F: CAACTATGTGGGGGACTCGG |
|  | R: TGGCTCTGGGCACATACTTG |
| VEGF-Rat | F: GCACTGGACCCTGGCTTTACT |
|  | R: AACTTCACCACTTCATGGGCTTT |
| CD31-Rat | F: GCTCTTCTACACGCCAACCT |
|  | R: TCATCTTGACTTCGTTGCCA |
| TNF-α-Rat | F: CATCTTCTCAAAATTCGAGTGACAA |
|  | R: TGGGGGTTGTGAGTGTGAGAT |
| IL-6-Rat | F: CTGCAAGAGACTTCCATCCAG |
|  | R: TCCACGATTTCCCAGAGAAC |
| TGF-β-Rat | F: ATTCCTGGCGTTACCTTGG |
|  | R: CCTGTATTCCGTCTCCTTGG |
| IL-10-Rat | F: AGTCTTCACCTGCTCCACTC |
|  | R: TCTCCATGCTGGGTCATCTT |

**TableS3 Categories of antibody**

| Antibody | Company | Cat. ID |
| --- | --- | --- |
| OCN | Servicebio | ER1919-20 |
| ALP | Servicebio | ab307726 |
| COL-1 | Servicebio | GB114197 |
| RUNX2 | Servicebio | GB13264 |
| β-actin | Servicebio | GB11001 |
| CD31 | Servicebio | GB11063-2 |
| VEGF | Servicebio | GB11034B |
| S100 | Servicebio | GB15359 |
| NF-200 | Servicebio | GB12143 |
| MEK | Servicebio | GB15304 |
| P-MEK | Servicebio | GB115603 |
| ERK | Servicebio | GB11560 |
| P-ERK | Servicebio | GB11004 |
| β-actin | Servicebio | GB15003 |
| PI3K | Servicebio | GB11525 |
| P-PI3K | Affinity | AF3242 |
| AKT | Servicebio | GB15689 |
| P-AKT | Servicebio | GB150002 |
| NDUFS5 | Affinity | AF9124 |
| UQCR10 | Three Eagles | 17779-1-AP |
| COX6C | Affinity | AF0762 |
| HRP, Goat Anti-Rabbit | Servicebio | GB23303 |
| HRP, Goat Anti-Mouse | Servicebio | GB23301 |
